# Evaluating musculoskeletal imaging communication interventions using behavioural science: a scoping review using the COM-B model

**DOI:** 10.1136/bmjopen-2024-085807

**Published:** 2025-04-09

**Authors:** Edward Kirby, Andrew MacMillan, Andrew Brinkley, Bernard X W Liew, Andrew Bateman

**Affiliations:** 1 School of Sport, Rehabilitation and Exercise Sciences, University of Essex, Colchester, UK; 2 Outpatient Physiotherapy, Connect Health Ltd, Newcastle upon Tyne, UK; 3 University of Essex, Colchester, UK

**Keywords:** Diagnostic Imaging, Behavior, Musculoskeletal disorders, Systematic Review

## Abstract

**Objectives:**

Clinicians and patients have been found to attribute musculoskeletal (MSK) pain to normal age-related changes seen on imaging, which can negatively impact patient outcomes and increase healthcare costs. While some studies have tested interventions to improve how MSK imaging findings are communicated, their impact has been limited. Applying a behavioural science framework has the potential to identify the rationale and target of these interventions to inform future intervention design—an analysis that has not yet been conducted. This study aims to identify the Behaviour Change Techniques (BCTs), the behavioural targets and the theoretical basis of interventions seeking to affect the communication of MSK imaging.

**Design:**

Scoping review using the Capability, Opportunity, Motivation - Behaviour (COM-B) model.

**Data sources:**

Searches of MEDLINE, EMBASE, CINAHL, AMED and PsycINFO from inception to 9 February 2024.

**Eligibility criteria for selecting studies:**

We included studies that have developed or evaluated interventions which target the communication of MSK imaging findings. Interventions targeting both patients and clinicians were included. Experimental and quasi-experimental study designs were included, and studies that focused on serious or specific known causes of MSK pain were excluded.

**Data extraction and synthesis:**

Two independent authors extracted study participant data and intervention details. A theory of behaviour called the COM-B model was used to map the BCTs and behavioural components targeted by studies.

**Results:**

We identified 11 studies from 2486 studies in our electronic search. 11 different BCTs were identified across 11 studies. The most common techniques were framing/reframing (nine studies), adding objects to the environment (eight studies), incompatible beliefs (seven studies) and avoidance/reducing exposure to cues for the behaviour (four studies). Only two studies (feasibility studies) used behavioural theory to guide their intervention design. While one study showed a large effect, most interventions had little to no impact on pain, disability, or fear over time.

**Conclusion:**

This review highlighted a lack of studies targeting clinician knowledge and the provision of high-quality patient resources about the nature of MSK pain, even though the broader literature identifies both as enablers of effective health communication. Additionally, the absence of a theory-informed design likely resulted in attempts to reassure patients about normal age-related imaging findings without providing an alternate, more coherent explanation for symptoms. Future interventions should focus on enhancing clinician psychological capability (knowledge) as well as clinician and patient reflective motivation (beliefs) to enable more helpful explanations of MSK symptoms. The key challenge for future interventions will be achieving these aims in a way that is effective, consistent and practical.

**Trial registration details:**

Open Science Framework (https://doi.org/10.17605/OSF.IO/ECYS8).

**STRENGTHS AND LIMITATIONS OF THIS STUDY**

- Comprehensive search strategy using a range of databases, hand searching and rigorous article screening performed by two independent authors and a third author to reconcile discrepancies.
- Studies using techniques targeting the reporting of imaging may be embedded in broader management techniques and therefore may have been missed by this search strategy.
- Subjective decisions involved in categorising interventions according to the Behaviour Change Techniques Taxonomy version 1 (BCTTv1).
- A further iteration of the BCTTv1 called the Behaviour Change Technique Ontology has been published since the submission of this manuscript.

## Introduction

 Epidemiological data indicates that musculoskeletal (MSK) disorders, such as arthritis and back pain, were the leading cause of disability globally in 2019.^1^ In the UK, they are estimated to affect 20 million people in 2023, accounting for more than 22% of the nation’s total burden of ill health. The rising disability is matched by rising costs, with the National Health Service spending £4.7 billion on MSK conditions in 2013–2014, which is expected to rise to £6.3 billion in 2023–2024.^2^ A reason cited for these rising costs and disability levels is the perpetuation of negative or unhelpful beliefs about the nature of MSK conditions.^3^,^5^

The belief, based on the biomedical model of health, that abnormalities visible on imaging are causally linked with pain^6^ has been associated with fear-avoidance behaviours,^7^ reduced physical activity^8^ and over-reliance on medical interventions,^9^ which in turn contribute to prolonged disability,^5^ higher healthcare utilisation^9^ and increased economic burden.^3^ The belief that abnormalities visible on imaging are causally linked with pain has been challenged since epidemiological studies discovered that imaging findings such as disc degeneration in the spine,^10^ degenerative cartilage tears in the knee^11^ and tendon tears in the shoulder^12^ are equally common in asymptomatic populations. These findings are therefore now considered to be normal age-related findings or ‘incidental findings’ with little or no association with pain. There is strong evidence that the practice of understanding and communicating these normal age-related findings in terms of pathological change has several negative consequences. These include: increased fear of movement (FoM),^13^ perception of a poorer prognosis,^14^ reduced confidence in non-surgical management,^15^ withdrawal from valued life activities,^16^ impaired general health outcomes,^17^ increased costs due to time off work^18^ and unnecessary imaging and treatments of low therapeutic value such as injections and surgery.^18 19^ Clinical guidelines recommend identifying and addressing such unhelpful beliefs in patients presenting with MSK symptoms.^20^ Unfortunately, however, evidence exists that healthcare professionals (HCPs) continue the unhelpful and inaccurate practice of framing normal age-related findings as pathological.^13 21 22^

The reasons why clinicians continue to communicate normal age-related MSK findings as pathology have been investigated in several qualitative studies. A lack of clinician knowledge about what constitutes an abnormal finding,^23 24^ entrenched biomedical beliefs among HCPs,^21 23 25^ a lack of clinician skill and confidence to communicate alternate psychosocial explanations for pain,^26^,^28^ the reluctance to challenge the patient’s current beliefs about the cause of pain,^29^ perception of reduced patient health literacy^29^ and a lack of time to discuss psychosocial factors^29^,^31^ have all been identified.

Once unhelpful behaviours are identified, evidence suggests that studies are more effective when they use a systematic approach to select interventions to target these behaviours.^32^,^34^ This study aims to determine whether such a structured process has been applied to the issue of unhelpful imaging communication. Additionally, the review will reanalyse previous interventions through the lens of a behavioural theory to assess whether suitably targeted techniques have already been designed, even if unintentionally.^35 36^ An intuitive and widely accepted behavioural theory; the Capability, Opportunity, Motivation - Behaviour (COM-B) model,^37^ has been used to assist the design of effective, feasible and affordable interventions targeting various health behaviours, including studies on weight loss,^38^ physical activity studies^39^ and smoking cessation studies.^40^ The theory posits that a person must have Capability, Opportunity and Motivation to perform and maintain a particular health behaviour.^37^ Each of these components is further divided into two, where capability can refer to the physical capability (eg, strength) and/or psychological capability (eg, knowledge) to perform the behaviour, the social opportunity (eg, interpersonal influences) and/or physical opportunity (eg, time and resources), and the automatic motivation (eg, desire) and/or reflective motivation (eg, beliefs about what is good). Specific Behaviour Change Techniques (BCTs) used in interventions have been found to target particular COM-B components.^41^,^43^ For instance, a BCT which provides information about health consequences will target patients’ reflective motivation (COM-B component) by providing knowledge. In addition to guiding intervention design, the simplicity of the COM-B model makes it an ideal framework for reanalysing existing interventions to assess whether the key behavioural components influencing behaviour have been effectively addressed.^35^

Given the rising burden of MSK disability and health system costs,^1 2^ alongside evidence of harmful MSK imaging communication practices,^13 22 44^ effective interventions which are systematically designed using behavioural science are overdue.^34^ This study seeks to ascertain whether interventions in the field of MSK imaging communication have used systematic intervention design frameworks, which BCTs have been tried thus far, their behavioural targets and their effectiveness. This information will be used to refine existing interventions or design new interventions targeting more influential drivers of behaviour. A preliminary search of PROSPERO, Open Science Framework, MEDLINE, the Cochrane Database of Systematic Reviews and the JBI Evidence Synthesis indicates that no study has attempted to map the BCTs implemented to affect the communication of MSK imaging findings or the theory underpinning these interventions to date.

## Aims and objectives

The aim of this scoping review is to examine how BCTs are used in interventions designed to improve the communication of MSK imaging findings. Specifically, the review seeks to:

- Examine whether studies follow a structured, systematic approach when developing communication interventions.
- Assess whether included studies explicitly use formal behavioural theories to guide intervention design.
- Identify which BCTs have been used in interventions, categorising them using a recognised taxonomy.
- Determine which behavioural components (Capability, Opportunity, Motivation) these BCTs target.
- Explore potential links between specific BCTs and the effectiveness of interventions.

## Methods

The scoping review was conducted in accordance with the JBI methodology for scoping reviews^45^ and the Preferred Reporting Items for Systematic reviews and Meta-Analyses (PRISMA) extension for Scoping Reviews.^46^ The protocol was registered with the Open Science Framework (https://doi.org/10.17605/OSF.IO/ECYS8) and published in BMJ Open https://bmjopen.bmj.com/content/13/11/e072150.long.

### Information sources and search strategy

The search was developed using a three-step strategy.^45^ First, an initial limited search of MEDLINE (PubMed) and CINAHL (EBSCO) was undertaken using a combination of three search terms derived from the target population (adults with MSK conditions), concept (communication of imaging) and context (experimental studies in any setting worldwide). Second, the identified keywords, text words and index terms contained in the titles and abstracts of the 10 most relevant articles were used to populate a full list of search terms. The search terms were adapted to each database (MEDLINE, EMBASE, AMED, CINAHL and PsycINFO) and run on 8 December 2022 (see online supplemental table 1). Third, the reference lists of all articles and reports included in the review were manually searched to identify additional studies. A grey literature search was not conducted, as one of the aims was to determine whether interventions had incorporated systematic intervention design and/or behavioural theory, which is difficult to ascertain and unlikely to be fully detailed in unpublished sources. A specialist healthcare librarian reviewed and refined the search strategy and terms. Additionally, four experts in the field of imaging and low back pain (LBP) were contacted via email to identify any studies that might have been overlooked. A final search was conducted by the first author on 9 February 2024, which did not yield any additional eligible studies.

### Eligibility criteria

The inclusion criteria, developed from the target population, concept and context, were:

- Experimental studies.
- Studies relating to adults with MSK conditions.
- Studies including interventions which targeted the communication of imaging results. Interventions targeting patients, clinicians or both will be included.

Experimental and quasi-experimental study designs including randomised controlled trials, non-randomised controlled trials, before and after studies, interrupted time-series studies, cluster randomised trials, non-randomised cluster trials, controlled and uncontrolled before–after studies, cross-sectional and feasibility studies were included. The review was not limited by publication year. Studies that include multiple interventions or broad treatment approaches were included if it was possible to isolate specific BCTs intended to affect the communication of MSK imaging.

The review excluded studies that focused on serious or specific known causes of MSK pain such as fracture, malignancy, infection and inflammatory arthritis.

### Study selection

After completing the search, all identified citations were uploaded into RefWorks (ProQuest LLC, Michigan, USA), where duplicates were removed. A pilot test of the first 20 articles alphabetically was conducted by two reviewers (EK and AM) together, to ensure the ongoing consistency in applying the inclusion and exclusion criteria. Titles and abstracts were then independently reviewed by the same two reviewers for inclusion in the full-text review. No additional information was needed to clarify eligibility at this point. Disagreements were resolved through discussion or by consulting a third reviewer (BXWL). Full texts of potentially relevant articles were retrieved and assessed in detail against the inclusion criteria by the same two reviewers. Reasons for excluding studies at the full-text screening stage were systematically coded and documented (see online supplemental table 2). Any disagreements during this stage were resolved through discussion or consultation with a third reviewer (BXWL). A final list of included studies was compiled, and the reference lists of these studies were manually searched by two reviewers (EK and AM) to identify any relevant missing studies.

### Data extraction

Two reviewers (EK and AM) independently extracted the intervention study characteristics which included: publication year, country, setting, study design, patient numbers (n), MSK area, outcome measures and summary of results (see online supplemental table 3). The Template for Intervention Description and Replication (TIDieR)^47^ (see online supplemental table 4) was used to describe each intervention. Section 3 on the template—‘Why - describe the use of any rationale, theory or goal of the elements essential to the intervention’ was used to extract evidence of theory utilisation. One author was successfully contacted to clarify the intended target of their intervention.^48^

Data were also collected on pain, disability and FoM at short-term, medium-term and long-term follow-ups to enable comparison. For each study, the sample size (n), mean values, and SD of the relevant outcomes were extracted.

### Data synthesis

Two reviewers (EK and AM) independently categorised each study intervention and linked them to their behavioural target.^37^ This process was enabled by two existing tools designed for this purpose. Categorisation of interventions was performed using the Behaviour Change Techniques Taxonomy version 1 (BCTTv1), a recognised method of categorising BCTs, which can be used as a method for specifying, evaluating and implementing complex behavioural change interventions.^49^ A pilot test was conducted by two reviewers (EK and ABr) on three studies, using the BCTTv1, to ensure consistency of categorisation. Notes and reasoning processes from this pilot were used to develop a coding manual which was used as a reference for future coding (see online supplemental table 5).

Additionally, to understand the process through which BCTs affect behaviour, The Theory and Techniques tool (TATT) (available at: https://theoryandtechniquetool.humanbehaviourchange.org/tool) was used to link BCTs with their behavioural target.^49^ The TATT is also able to determine the strength of the link based on a triangulation study,^41^ a literature synthesis^43^ and an expert consensus study.^42^ Where available, the p values indicating the strength of the link have been included (see online supplemental table 7). Any disagreement in categorisation was resolved through discussion or resolution by a third reviewer (AM)

The stated theoretical influences of studies were extracted and analysed according to the Painter criteria which categorise the use of theory as: (1) informed by, (2) testing or (3) creating theory.^50^ The extent of use of theory was determined by assessing the stated theoretical underpinning within the manuscript and evaluating the congruence with the study outcome measures by two reviewers (EK and AM). Evaluation of the quality of included studies was not performed as this was not an objective of the review.

Finally, forest plots to visualise effect sizes were developed, allowing for a comparison of intervention impact on pain, disability and FoM across different follow-up periods (see online supplemental figure 2). The ‘meta’ package in R software was used to calculate the standardised mean difference (SMD) (where available) for each study, each subtask within the study and for each pairwise comparison between pain and asymptomatic conditions.^51 52^

### Patient and public involvement

Patients with experience of receiving MSK imaging reports were invited to participate in individual sessions initially by advertising in person and using study posters and flyers within radiology departments and GP surgeries. These initial sessions sought to explore peoples’ experience of imaging report communication and the ways that this could be improved, such as the setting, personnel involved and resources that would be helpful to them. These meetings highlighted clinical behaviours that were discordant with patient preference and best practice.^20^ Based on this information, it was deemed necessary to investigate the barriers to providing helpful communication of reports and the current review of whether existing interventions had targeted these. Further patient and public involvement group sessions are planned to discuss the results of this review and to have input into the design and implementation of further work seeking to improve the communication of MSK imaging findings.

## Results

The electronic search identified a total of 3841 citations. After 1261 duplicates were removed and 2486 records were excluded during title and abstract screening, 94 full-text articles were retrieved. One further eligible study was identified by searching the reference lists of the included articles. The full text of 95 articles was screened, and 84 articles were excluded, leaving 11 studies. The results of the search and the study inclusion process are presented in a PRISMA flow diagram^53^ (see online supplemental figure 1).

### Description of studies

Interventions within studies included the withholding of information from patients about their imaging results,^17^ adding prevalence information to results,^54^,^59^ rewording results^48 58 60 61^ and using resources to teach patients about their condition.^58 59 62^ Full details of interventions are included in the TIDieR template (online supplemental table 4), with brief study descriptions included in online supplemental tables 6 and 7.

Nine of the studies were performed in the last 5 years (2018 to February 2024). Five studies were performed in the USA, four in Australia and one in India and China, respectively. Eight of the studies employed a randomised controlled design, which includes two feasibility studies. Three of the studies performed in Australia recruited members of the public without MSK pain and used hypothetical scenarios. Most patients across all studies were recruited within primary care (n=239 571). The study characteristics are presented in online supplemental table 3.

### Use of theory and/or systematic intervention design process

9 of the 11 studies in this review did not reference or incorporate an established theoretical framework to guide their intervention design, nor did they follow a systematic approach to developing their interventions. Furthermore, studies which shared similar interventions such as the addition of prevalence information did not share similar outcomes. Conversely, the two feasibility studies explicitly used recognised theoretical frameworks. Both studies used the Conceptual Change Model (CCM),^58 59^ which outlines a process including the confrontation of existing unhelpful beliefs and the introduction of new, more plausible concepts. The interventions used in one feasibility study^58^ clearly targeted common misconceptions about imaging (the association between imaging findings and pain) and introduced a new concept for the cause of pain (a response to perceived threat rather than tissue damage). Their outcome measures include ratings of pain intensity, disability and FoM, but do not evaluate patient beliefs about back pain, which is a key construct in the CCM.^63^ Most of the constructs of the CCM were, however, measured, and as such, the study was categorised as ‘testing theory’.^50^ The second feasibility study^59^ tested a Pain Science Education (PSE) programme, a treatment strategy informed by the CCM.^64^ They propose that in this context, PSE seeks to shift the meaning of participants’ pain from a direct correlate of tissue damage to that of a perception of threat affected by biological, psychological and social factors. The study measures FoM, self-efficacy, pain beliefs and pain neuroscience knowledge, and for this reason was also categorised as ‘testing theory’.^50^

### BCTs used

11 different BCTs were identified across all studies. Each study employed anywhere between 1 and 10 BCTs as part of their interventions with an average of 3.7 BCTs per study. The most common techniques were framing/reframing (nine studies), adding objects to the environment (eight studies), incompatible beliefs (seven studies) and avoidance/reducing exposure to cues for the behaviour (four studies). Online supplemental table 6 presents the BCTs identified in each study. The most comprehensive intervention was a feasibility study^58^ which employed 10 different BCTs, 9 of which targeted participants and 6 targeted clinicians through training and the provision of resources.

### COM-B targets

Online supplemental table 7 illustrates how these BCTs were mapped to their COM-B components.^49^ A total of three out of the six COM-B components; psychological capability, physical opportunity and reflective motivation, were targeted by the BCTs in the included studies. Three COM-B components, physical capability, social opportunity and automatic motivation, were not targeted by any studies in this review. Two feasibility studies targeted three COM-B components simultaneously^58 59^ and six studies targeted the psychological capability and reflective motivation components simultaneously.^54^,^5760 61^

### Capability

All 11 studies targeted psychological capability, with two feasibility studies^58 59^ targeting the psychological capability of both clinicians and participants. One of these feasibility studies trained the study clinicians in a novel imaging interpretation framework^58^ and another trained their study clinicians in a PSE programme,^59^ both of which involved ‘instruction on how to perform the behaviour’, ‘demonstration of the behaviour’ and ‘behavioural practice’. In addition to targeting clinicians, these studies targeted the capabilities of study participants by imparting information about the benefits of movement and reassuring participants that ‘activity and exercise are safe and do not lead to further structural damage’.^58^ In doing so, they employed the BCT ‘information about health consequences’. One of these feasibility studies also attempted to regulate behaviour by sending text message prompts to participants encouraging them to read information and prepare their exercise plans, which was categorised as ‘action planning’.^58^

### Opportunity

Eight studies included BCTs which sought to affect the physical opportunity to communicate imaging findings by either ‘avoiding or reducing exposure to cues for the behaviour’^17 48 60 61^ or ‘adding objects to the environment’.^48 58 59 62^ Interventions avoiding and reducing exposure to imaging reports targeted this component by blinding participants and clinicians to the imaging results^17^ or altering the content of imaging results.^60 61^

### Motivation

Nine studies included BCTs that targeted the reflective motivation of patients. One feasibility study^58^ provided a video containing information about MSK pain, presented by a pain expert (‘credible source’), which was considered to have an additional influence on beliefs beyond the information itself.

Patient and clinician attitudes and beliefs were also targeted by ’reframing techniques’ such as rewording reports,^60 61^ re-interpreting reports^58^ and adding information about the prevalence of the findings in the pain-free population.^54^,^57^ The reflective motivation of solely clinicians was also targeted in two studies via the demonstration of a novel imaging communication framework.^57 58^ The knowledge and skills provided in these studies were considered to have affected clinicians’ reflective motivation in addition to psychological capability as they targeted clinician beliefs about their capabilities and the consequences of helpful, non-threatening explanations for MSK pain.

### Intervention effectiveness

The three forest plots (online supplemental figure 2) present the SMDs and CIs for pain, disability and fear outcomes at different time points (<6 weeks, 3–6 months and>1 year) across studies with relevant outcome measures.

#### Pain

The effects of interventions on pain reduction were inconsistent. The study by Rajasekaran *et al*^60^ showed a significant reduction in pain at<6 weeks (SMD: −2.84, 95% CI: −3.70 to −1.98). Other studies showed small, non-significant effects, with SMDs ranging from −0.20 to −0.04, suggesting minimal to no difference between intervention and control groups in pain levels over time.

#### Disability

The interventions had little to no effect on disability across time points. The SMD values were small and non-significant (−0.18 to 0.14), with CIs crossing zero, indicating no clear difference between groups.

#### Fear

The impact of interventions on reducing fear was inconsistent. Most studies at<6 weeks showed small, non-significant effects (SMD: −0.06 to 0.13). At 3–6 months, Karran *et al*^58^ showed a moderate but non-significant increase in fear (SMD: 0.52, 95% CI: −0.23 to 1.26). Long-term effects (>1 year) were negligible (SMD: 0.09, 95% CI: −0.16 to 0.34).

Overall, the results suggest that while one study found a large effect, most interventions had minimal or no impact on pain, disability or fear over time.

## Discussion

### Systematic design and use of behavioural theory

One of the core recommendations of the National Institute for Health and Care Research and Medical Research Council framework for the systematic design of complex interventions is for a programme theory, underpinned by evidence, to be developed at the beginning of a research project, which describes how an intervention is expected to lead to its outcomes.^65^ Two feasibility studies included in this review explicitly used a learning theory called the CCM to develop their interventions.^57 58^ This model posits that a person must develop dissatisfaction with their current beliefs before a new, more coherent, plausible and helpful concept can replace it.^63^ The interventions developed in the two studies were directly influenced by the CCM, where the prior knowledge and beliefs of the learner and their motivation and capability to engage with new information were considered. They then developed a range of information resources and active learning strategies that targeted patient and clinician capability and motivation to enable patients to reconceptualise their MSK pain. The studies^58 59^ were designed to explore the acceptability, feasibility and implementation of the interventions rather than test their effectiveness. As such, they lack the statistical power (n=20 and n=10 in the experimental groups) and rigorous design required to draw definitive conclusions about efficacy, limiting the validity and generalisability of their findings.

Several studies included in this review evaluated interventions targeting behaviours without explicitly utilising behavioural science. Four studies challenged the biomedical basis of patients’ pain by providing prevalence statements and reassurance.^54^,^57^ These studies then evaluated constructs such as FoM,^66^ back-related perceptions^67^ and healthcare utilisation.^54 55 57^ By applying interventions which challenge patients’ current biomedical beliefs and then measuring belief and behavioural outcomes, these study designs inadvertently evaluated the constructs of several relevant health behaviour theories (the fear avoidance model,^66^ the common-sense model of health^67^ and the theory of misdirected learning^68^).

By not explicitly using a relevant behavioural theory as a framework, authors may have missed the opportunity to apply more targeted BCTs,^35 69^ potentially prolonging intervention development^33^ and limiting the insights gained from their results.^33 69 70^ The limited range of interventions in this review with studies targeting only half of all the possible COM-B components (psychological capability, physical opportunity, reflective motivation) is a symptom of the lack of behavioural theory utilisation. The publication of the MRC framework for developing complex interventions in 2006^71^ promoted more systematic and targeted intervention design. Awareness of this guidance has increased over time, and it was updated in 2021.^65^ While it is feasible that the study published in 2008^17^ did not fully benefit from this framework, the lack of theoretical integration in the other studies is more difficult to justify.

Some of the notable omissions in interventions in this review include persuasion, incentivisation, coercion and enablement.^37^ Evidence suggests that incentivisation can improve exercise adherence and support weight loss,^72 73^ while persuasion, particularly through positively framed messages, has been shown to influence attitudes and behaviours related to physical activity.^74 75^ These strategies, which are well-documented within broader behavioural theories and intervention design frameworks like the COM-B model^37^ could be relevant for improving MSK imaging communication.

### Psychological capability

Providing knowledge to patients (psychological capability) was the most targeted intervention in this review, though it was often limited to basic prevalence information.^54^,^57^ In contrast, two studies implemented comprehensive clinician training programmes,^58 59^ recognising the pivotal role of healthcare providers in shaping patient beliefs.^21 25 44 76^ Clinician studies demonstrating increased proficiency when performing newly acquired communication and management skills have used nine workshops in cognitive functional therapy,^77^ an 8-day university course,^78^ a 7-hour physiotherapy workshop^79^ and a 10-session training programme supplemented by ongoing supervision and online resources.^80^ Conversely, brief training programmes (1–2 sessions) result in incomplete adoption of biopsychosocial approaches,^81^,^85^ with clinicians mixing new skills with pre-existing biomedical approaches^86^ and overestimating their subsequent application of biopsychosocially informed practice.^86 87^ This suggests that effective conceptual change interventions are likely to require structured, theory-driven education with opportunities for practice and reinforcement. Despite this, all but two studies provided only brief information^54^,^57^ or reworded reports.^48 60 61^

Health literacy plays a crucial role in how patients understand and act on health information.^88^,^91^ Researchers have successfully mitigated the effect of health literacy by providing translated resources, presenting essential information by itself, using plain language, substituting textual content with graphical displays and adding video to verbal narratives.^92^,^94^ Only two studies in the current review, however, used multiple formats (posters, face-to-face and online content) to facilitate learning^58 59^ and none of the content and format of interventions in the review were designed to be adaptable to patients with variable psychological capabilities. Despite most interventions targeting psychological capability, interventions targeting patients provided limited uncontextualised information and only feasibility studies provided training to help clinicians communicate helpful imaging information more proficiently. This may explain the small or negligible effects on pain, disability and FoM, highlighting the need for more comprehensive and theory-driven strategies.

### Reflective motivation

Reflective motivation refers to an individual’s belief in a behaviour’s feasibility and benefit.^37^ Altering the reflective motivation of patients is central to relevant health behaviour theories such as the Fear-Avoidance model,^66^ the Common-Sense model of health^67^ and the Theory of Misdirected Learning.^68^ These theories posit that changing how patients perceive their symptoms can, in turn, shift their views on management, driving positive behavioural change. Successful management approaches which focus on reconceptualisation, such as PSE and Cognitive Functional Therapy, use varied and adaptable methods to identify and target existing patient beliefs and emotions before developing an alternate coherent and plausible explanation for their MSK pain.^95^,^100^ Such strategies have been associated with increases in beneficial health behaviours^101 102^ and improvements in pain and disability outcomes.^95^ This review identified two feasibility studies which considered the existing beliefs, cognitions and emotions of the patient before providing an alternate more plausible explanation to enable reconceptualisation of their pain.^58 59^ Without the provision of an alternative, more plausible explanation for their symptoms, patients have been found to struggle to integrate new information into their existing beliefs,^63 67 103^ which may explain why studies providing solely prevalence information demonstrated small effect sizes.^54^,^57^ One intervention which resulted in a large reduction in pain used an alternate method of ‘clinical reporting’ which eliminated terms causing concern and anxiety to patients without losing scientific clarity.^48 60^ This intervention, which was not reported in detail, would need to be clarified, standardised and further validated in larger, more rigorous trials before conclusions can be drawn about its effectiveness.

### Physical opportunity

The increased time burden of delivering alternate biopsychosocial explanations for imaging findings, compared with biomedical explanations, has been identified as a barrier to clinicians adopting this approach in several studies.^104 105^ The lack of adequate time to enable clinicians to effectively deliver a biopsychosocially informed explanation for MSK conditions has also been highlighted in surveys^30^ and qualitative studies.^29 106^ Only one study in the current review considered this when designing their intervention and provided clinicians with the opportunity of an additional 10 min to deliver a novel biopsychosocially informed imaging interpretation intervention.^58^ However, as a feasibility trial, it provides no empirical evidence that dedicating extra time to explaining imaging findings improves patient outcomes. Studies have found a small effect on prescription and blood pressure monitoring when General Practitioner (GP) consultation length was increased,^107^,^109^ but no change in patient satisfaction.^107 109^ At present, little evidence exists to suggest that extended clinical consultation time has any effect on productivity outcomes. Extending consultation time to explain imaging findings is therefore unlikely to be acceptable to commissioners of overburdened health systems without associated evidence of benefit to patients and the healthcare system.

In one study included in this review, patients and clinicians in the intervention group were blinded to their lumbar MRI results and compared with a control group, who were provided with a standard radiology report which was conveyed to patients using a standardised form.^17^ Standard radiology reports are written using medical terminology^110^ which is not comprehensible to the general public.^111^ Indeed, one study highlighted that fewer than one-third of general practitioners were entirely confident of their comprehension of MRI reports.^112^ The provision of technical, uncontextualised reports, as seen in the control arm of eight studies included in this review,^1748 54^,^57 60 61^ is an increasingly common situation in clinical practice where the physical opportunity to view imaging reports has been enabled by recent improvements in health technologies. Increasingly, patients are accessing radiology reports directly via online health portals^113 114^ with all GP surgeries in England now mandated to provide patients with online access to new information as it is added to their health record.^115^ Increased access to certain types of medical records has been shown to increase patient satisfaction, enable patients to be involved in health decisions and reduce the demand on GP practices to inform patients of results.^116 117^ However, the presentation of incidental findings in non-MSK imaging, without specialist interpretation and reassurance has been shown to negatively affect stress levels and quality of life.^118 119^ The increasingly sensitive imaging techniques,^120^ radiology reports containing incomprehensible medical terminology^61 121^ and the low quality of information in the public domain^122 123^ all increase the possibility for harmful misinterpretation of normal age-related findings. One study^17^ included in this review limited the physical opportunity of patients and clinicians to view their radiology reports to avoid the potential anxiety and misunderstanding that can arise from providing uncontextualised results. However, the intervention had no effect on pain, disability and fear of movement in the short- and long-term. When considering the CCM, comparing the absence of information with unintelligible information is unlikely to produce a meaningful effect. Only when reports are replaced with a plausible, helpful explanation for pain is a positive impact likely to be observed.

Three studies included in this review also targeted the physical opportunity component of COM-B by providing additional resources to be used within the clinical encounter. These included the addition of information resources such as books^58 59^ and the use of models.^62^ One of these studies provided the opportunity to access a resource outside of the clinical encounter in the form of an infographic supporting the reconceptualisation of imaging findings.^58^ Several studies investigating patients experience of LBP diagnosis have found that patients independently seek additional information about their condition after a clinical encounter.^124 125^ Unfortunately, this is commonly in the form of inaccurate or poor-quality online information,^122 123^ which is often not consistent with guidelines.^126^ The provision of supplementary high-quality information which complements the information provided within the clinical encounter not only increases patient satisfaction^127^ and comprehension of their condition,^128 129^ but also provides a further opportunity for conceptual change. The lack of BCTs providing the physical opportunity to access high-quality information resources relating to imaging findings in this review is therefore conspicuous.

As part of the rapidly advancing field of behaviour change research, a further iteration of the BCTTv1 called the Behaviour Change Technique Ontology (BCTO) has been published since the submission of the manuscript. The BCTO includes more BCTs, more detail and amendments based on feedback. This study would likely have been improved by the use of this framework and future research should consider applying this updated taxonomy to enhance the accuracy and consistency of behavioural coding in imaging communication interventions. A further limitation of this study is the potential omission of broader MSK imaging communication interventions, embedded within broader treatment or management interventions. To ensure that imaging communication approaches were not omitted, the search strategy in this review included the names of recognised biopsychosocial treatment models such as pain science education and cognitive functional therapy, alongside manual reference list searches. Studies which did not specifically target imaging communication were not included, which may have led to the omission of some pertinent communication interventions. However, the discrete point of imaging communication represents a key opportunity to shape patient beliefs and influence their subsequent management choices. Interventions that do not specifically address this moment were deemed less applicable to future studies aiming to improve imaging-related communication and were thus not included.

Finally, the process of categorising interventions according to the BCTTv1 involved a degree of subjective judgement, which may have influenced the consistency of coding. Although efforts were made to mitigate this through the development of a coding manual and independent review of two reviewers, some variability is inherent in this approach.

## Conclusion

Many of the interventions identified in this review typically targeted one or two COM-B components with no overt design process or accepted theoretical basis for doing so. In contrast, two feasibility studies used learning theory to design an intervention which included BCTs targeting multiple COM-B components including the potentially important components of clinician and patient psychological capability and reflective motivation. The review highlighted a scarcity of studies targeting clinician knowledge and the provision of high-quality patient resources, despite evidence in the wider literature that these are barriers to helpful imaging reporting behaviours.^130^ Further to this, the lack of theory-informed design is likely to have led to attempts to reconceptualise patient problems without the important step of providing an alternate, coherent, plausible and helpful concept for MSK pain.^63 67^ Future interventions should consider the targeting of clinician psychological capability (knowledge) and clinician and patient reflective motivation (beliefs) to enable coherent non-threatening representations of symptoms and the physical opportunity to access high quality, coherent and non-threatening information. The challenge for future interventions will be to achieve this in a way that remains feasible within overburdened health systems while also ensuring a consistent approach across healthcare services, accounting for the different ways patients access and receive imaging information.

## Supplementary material

10.1136/bmjopen-2024-085807 online supplemental file 1

## Data Availability

All data relevant to the study are included in the article or uploaded as supplementary information.
