# Supplementary material for: Evaluating musculoskeletal imaging communication interventions using behavioural science: a scoping review using the COM-B model
Source: BMJ Open. 2025 Apr 9;15(4):e085807. doi: 10.1136/bmjopen-2024-085807 (PMC11987107; doi:10.1136/bmjopen-2024-085807)
Supplement: online supplemental file 1 [file bmjopen-15-4-s001.docx]

Supplementary Figure 1 – PRISMA Flowchart of studies


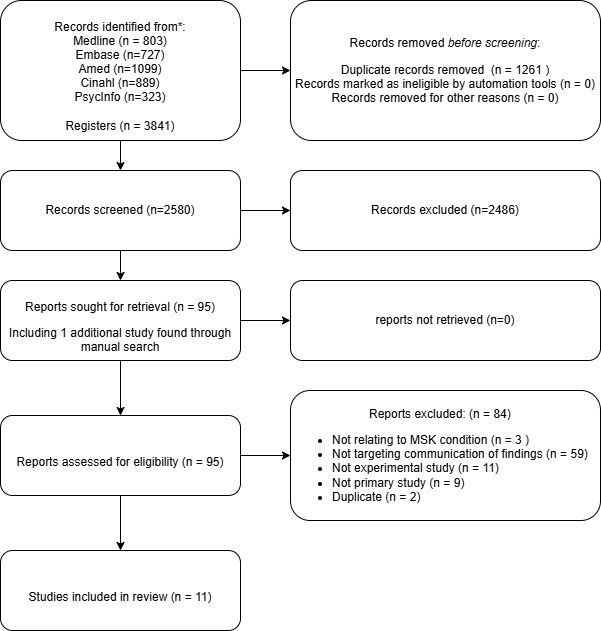


Supplementary table 1 – Search terms

|  | **MEDLINE** |
| --- | --- |
| 1. | exp Musculoskeletal Pain/ or musculoskeletal.mp. |
| 2. | exp Patellofemoral Pain Syndrome/ or exp Back Pain/ or exp Low Back Pain/ or exp Shoulder Pain/ or exp Neck Pain/ |
| 3. | osteoarthriti*.mp. or exp Osteoarthritis, Hip/ or exp Osteoarthritis/ or exp Osteoarthritis, Spine/ or exp Osteoarthritis, Knee/ |
| 4. | spondylosis.mp. or exp Spondylosis/ |
| 5. | (backache or lumbago or radicular or sciatica).mp. |
| 6. | ("leg pain" or "foot pain" or "heel pain" or "ankle pain" or "knee pain" or "patellofemoral pain" or "hip pain" or "arm pain" or "shoulder pain" or "subacromial pain" or "elbow pain" or "hand pain" or "wrist pain").mp. [mp=title, book title, abstract, original title, name of substance word, subject heading word, floating sub-heading word, keyword heading word, organism supplementary concept word, protocol supplementary concept word, rare disease supplementary concept word, unique identifier, synonyms] |
| 7. | exp chronic pain/ or "chronic pain".mp. or "persistent pain".mp. [mp=title, book title, abstract, original title, name of substance word, subject heading word, floating sub-heading word, keyword heading word, organism supplementary concept word, protocol supplementary concept word, rare disease supplementary concept word, unique identifier, synonyms] |
| 8. | exp Humans/ |
| 9. | exp Animals/ |
| 10. | 8 and 9 |
| 11. | 9 not 10 |
| 12. | "diagnostic imaging".mp. or exp Diagnostic Imaging/ |
| 13. | exp Radiography/ or radiograph*.mp. |
| 14. | IMAGING.mp. or exp Magnetic Resonance Imaging/ or "magnetic resonance".mp. or MRI.mp. |
| 15. | exp Radiology/ or exp Radiologists/ or RADIOLOG*.mp. or exp Radiology Information Systems/ |
| 16. | Tomography, X-Ray Computed/ or "COMPUTED TOMOGRAPHY".mp. |
| 17. | CT.mp. |
| 18. | (ultrasound or ultrasonograph*).mp. [mp=title, book title, abstract, original title, name of substance word, subject heading word, floating sub-heading word, keyword heading word, organism supplementary concept word, protocol supplementary concept word, rare disease supplementary concept word, unique identifier, synonyms] |
| 19. | exp X-Rays/ |
| 20. | X?RAY.mp. |
| 21. | exp Education, Medical/ or exp Interprofessional Education/ or exp Patient Education Handout/ or exp Patient Education as Topic/ or exp Health Education/ |
| 22. | exp Health Communication/ |
| 23. | "HEALTH COMMUNICATION".mp. [mp=title, book title, abstract, original title, name of substance word, subject heading word, floating sub-heading word, keyword heading word, organism supplementary concept word, protocol supplementary concept word, rare disease supplementary concept word, unique identifier, synonyms] |
| 24. | "REPORTING STRATEG*".mp. |
| 25. | (EPIDEMIOLOGIC* adj2 (DATA or INFORMATION)).mp. [mp=title, book title, abstract, original title, name of substance word, subject heading word, floating sub-heading word, keyword heading word, organism supplementary concept word, protocol supplementary concept word, rare disease supplementary concept word, unique identifier, synonyms] |
| 26. | (PREVALENCE adj2 INFORMATION).mp. [mp=title, book title, abstract, original title, name of substance word, subject heading word, floating sub-heading word, keyword heading word, organism supplementary concept word, protocol supplementary concept word, rare disease supplementary concept word, unique identifier, synonyms] |
| 27. | (PAIN adj2 EDUCATION).mp. [mp=title, book title, abstract, original title, name of substance word, subject heading word, floating sub-heading word, keyword heading word, organism supplementary concept word, protocol supplementary concept word, rare disease supplementary concept word, unique identifier, synonyms] |
| 28. | ("pain neurobiology" or "pain neurophysiology").mp. [mp=title, book title, abstract, original title, name of substance word, subject heading word, floating sub-heading word, keyword heading word, organism supplementary concept word, protocol supplementary concept word, rare disease supplementary concept word, unique identifier, synonyms] |
| 29. | Patient Education as Topic/ or PSYCHOEDUCATION*.mp. |
| 30. | (COMMUNICAT* adj5 ("IMAGing FINDINGs" or "IMAGing RESULTs" or "imaging report" or " MRI findings" or "MRI results" or "MRI REPORT*" or "X-RAY findings" or "X-Ray results" or "X-Ray REPORT" or "CT findings" or "CT REPORT" or "CT results" or "RADIOLOG* FINDINGS*" or "radiolog* results" or "radiolog* report")).mp. [mp=title, book title, abstract, original title, name of substance word, subject heading word, floating sub-heading word, keyword heading word, organism supplementary concept word, protocol supplementary concept word, rare disease supplementary concept word, unique identifier, synonyms] |
| 31. | ((WRITTEN or PRINTED or ORAL) adj INFORMATION).mp. [mp=title, book title, abstract, original title, name of substance word, subject heading word, floating sub-heading word, keyword heading word, organism supplementary concept word, protocol supplementary concept word, rare disease supplementary concept word, unique identifier, synonyms] |
| 32. | (interpretation adj3 ("IMAGing FINDINGs" or "IMAGing RESULTs" or "imaging report" or " MRI findings" or "MRI results" or "MRI REPORT*" or "X-RAY findings" or "X-Ray results" or "X-Ray REPORT" or "CT findings" or "CT REPORT" or "CT results" or "RADIOLOG* FINDINGS*" or "radiolog* results" or "radiolog* report")).mp. [mp=title, book title, abstract, original title, name of substance word, subject heading word, floating sub-heading word, keyword heading word, organism supplementary concept word, protocol supplementary concept word, rare disease supplementary concept word, unique identifier, synonyms] |
| 33. | (REASSUR* adj3 ("IMAGing FINDINGs" or "IMAGing RESULTs" or "imaging report" or " MRI findings" or "MRI results" or "MRI REPORT*" or "X-RAY findings" or "X-Ray results" or "X-Ray REPORT" or "CT findings" or "CT REPORT" or "CT results" or "RADIOLOG* FINDINGS*" or "radiolog* results" or "radiolog* report")).mp. [mp=title, book title, abstract, original title, name of substance word, subject heading word, floating sub-heading word, keyword heading word, organism supplementary concept word, protocol supplementary concept word, rare disease supplementary concept word, unique identifier, synonyms] |
| 34. | (CONTEXTUALI* adj3 ("IMAGing FINDINGs" or "IMAGing RESULTs" or "imaging report" or " MRI findings" or "MRI results" or "MRI REPORT*" or "X-RAY findings" or "X-Ray results" or "X-Ray REPORT" or "CT findings" or "CT REPORT" or "CT results" or "RADIOLOG* FINDINGS*" or "radiolog* results" or "radiolog* report")).mp. [mp=title, book title, abstract, original title, name of substance word, subject heading word, floating sub-heading word, keyword heading word, organism supplementary concept word, protocol supplementary concept word, rare disease supplementary concept word, unique identifier, synonyms] |
| 35. | ((perception or perceive) adj3 ("IMAGing FINDINGs" or "IMAGing RESULTs" or "imaging report" or " MRI findings" or "MRI results" or "MRI REPORT*" or "X-RAY findings" or "X-Ray results" or "X-Ray REPORT" or "CT findings" or "CT REPORT" or "CT results" or "RADIOLOG* FINDINGS*" or "radiolog* results" or "radiolog* report")).mp. [mp=title, book title, abstract, original title, name of substance word, subject heading word, floating sub-heading word, keyword heading word, organism supplementary concept word, protocol supplementary concept word, rare disease supplementary concept word, unique identifier, synonyms] |
| 36. | (DIAGNOS* adj2 INFORMATION).mp. [mp=title, book title, abstract, original title, name of substance word, subject heading word, floating sub-heading word, keyword heading word, organism supplementary concept word, protocol supplementary concept word, rare disease supplementary concept word, unique identifier, synonyms] |
| 37. | (INTERVENTION* adj5 ("IMAGing FINDINGs" or "IMAGing RESULTs" or "imaging report" or " MRI findings" or "MRI results" or "MRI REPORT*" or "X-RAY findings" or "X-Ray results" or "X-Ray REPORT" or "CT findings" or "CT REPORT" or "CT results" or "RADIOLOG* FINDINGS*" or "radiolog* results" or "radiolog* report")).mp. [mp=title, book title, abstract, original title, name of substance word, subject heading word, floating sub-heading word, keyword heading word, organism supplementary concept word, protocol supplementary concept word, rare disease supplementary concept word, unique identifier, synonyms] |
| 38. | BIOPSYCHOSOCIAL.mp. |
| 39. | (behavio?r adj2 intervention).mp. |
| 40. | "behavio?r change".mp. |
| 41. | exp cognitive behavioral therapy/ or "cognitive behavio?ral therapy".mp. |
| 42. | "cognitive functional therapy".mp. |
| 43. | exp Arthritis, Rheumatoid/ |
| 44. | exp Neoplasms/ or sarcoma*.mp. or osteosarcoma*.mp. or tumo?r*.mp. |
| 45. | exp Musculoskeletal Diseases/cn, su [Congenital, Surgery] |
| 46. | exp Central Nervous System/ |
| 47. | exp Central Nervous System Diseases/ |
| 48. | exp Dentistry/ |
| 49. | exp Tooth Diseases/ |
| 50. | 1 or 2 or 3 or 4 or 5 or 6 or 7 |
| 51. | 12 or 13 or 14 or 15 or 16 or 17 or 18 or 19 or 20 |
| 52. | 21 or 22 or 23 or 24 or 25 or 26 or 27 or 28 or 29 or 30 or 31 or 32 or 33 or 34 or 35 or 36 or 37 or 38 or 39 or 40 or 41 or 42 |
| 53. | 43 or 44 or 45 or 46 or 47 or 48 or 49 |
| 54. | 50 and 51 and 52 |
| 55. | 54 not 53 |
| 56. | 55 not 11 |
|  |  |
|  |  |
|  |  |
|  | **EMBASE** |
| 1. | exp Arthritis, Rheumatoid/ or exp Neoplasms/ or exp Central Nervous System/ or exp Central Nervous System Diseases/ or exp Dentistry/ or exp Tooth Diseases/ or sarcoma*.ti,ab. or osteosarcoma*.ti,ab. or tumour*.ti,ab. |
| 2. | exp Patient Education as Topic/ or patient education/ or "patient education".ti,ab. |
| 3. | exp health education/ or exp physical therapy education/ or exp chiropractic education/ or exp continuing education/ or exp clinical education/ or exp allied health education/ |
| 4. | exp Health Communication/ or "HEALTH COMMUNICATION".mp. |
| 5. | "REPORTING STRATEGIES".ti,ab. |
| 6. | (EPIDEMIOLOGIC* adj2 (DATA or INFORMATION)).ti,ab. |
| 7. | (prevalence adj2 (data or information)).mp. |
| 8. | (pain adj2 education).ti,ab. |
| 9. | exp psychoeducation/ or psychoeducation*.af. |
| 10. | (BEHAVIO?R adj2 INTERVENTION*).ti,ab. |
| 11. | exp cognitive behavioral therapy/ or "cognitive behavio?ral therapy".mp. |
| 12. | "behavio?r change".mp. |
| 13. | (COMMUNICAT* adj3 ("IMAGing FINDING*" or "IMAGing RESULTs" or "imaging report" or "MRI REPORT" or "MRI results" or "MRI finding*" or "X-RAY REPORT" or "X-ray results" or "x-ray finding*" or "CT REPORT" or "CT results" or "CT findings" or "RADIOLOG* results" or "radiolog* report" or "radiolog* FINDINGs")).ti,ab. |
| 14. | "pain neurobiolog*".ti,ab. |
| 15. | "PAIN NEUROPHYSIOLOG*".ti,ab. |
| 16. | ((WRITTEN or PRINTED or ORAL) adj INFORMATION).af. |
| 17. | (INTERPRETATION adj3 ("IMAGing FINDING*" or "IMAGing RESULTs" or "imaging report" or "MRI REPORT" or "MRI results" or "MRI finding*" or "X-RAY REPORT" or "X-ray results" or "x-ray finding*" or "CT REPORT" or "CT results" or "CT findings" or "RADIOLOG* results" or "radiolog* report" or "radiolog* FINDINGs")).mp. [mp=title, book title, abstract, original title, name of substance word, subject heading word, floating sub-heading word, keyword heading word, organism supplementary concept word, protocol supplementary concept word, rare disease supplementary concept word, unique identifier, synonyms] |
| 18. | (reassur* adj3 ("IMAGing FINDING*" or "IMAGing RESULTs" or "imaging report" or "MRI REPORT" or "MRI results" or "MRI finding*" or "X-RAY REPORT" or "X-ray results" or "x-ray finding*" or "CT REPORT" or "CT results" or "CT findings" or "RADIOLOG* results" or "radiolog* report" or "radiolog* FINDINGs")).mp. [mp=title, book title, abstract, original title, name of substance word, subject heading word, floating sub-heading word, keyword heading word, organism supplementary concept word, protocol supplementary concept word, rare disease supplementary concept word, unique identifier, synonyms] |
| 19. | (contextualis* adj3 ("IMAGing FINDING*" or "IMAGing RESULTs" or "imaging report" or "MRI REPORT" or "MRI results" or "MRI finding*" or "X-RAY REPORT" or "X-ray results" or "x-ray finding*" or "CT REPORT" or "CT results" or "CT findings" or "RADIOLOG* results" or "radiolog* report" or "radiolog* FINDINGs")).mp. [mp=title, book title, abstract, original title, name of substance word, subject heading word, floating sub-heading word, keyword heading word, organism supplementary concept word, protocol supplementary concept word, rare disease supplementary concept word, unique identifier, synonyms] |
| 20. | ((perception or perceive) adj3 ("IMAGing FINDING*" or "IMAGing RESULTs" or "imaging report" or "MRI REPORT" or "MRI results" or "MRI finding*" or "X-RAY REPORT" or "X-ray results" or "x-ray finding*" or "CT REPORT" or "CT results" or "CT findings" or "RADIOLOG* results" or "radiolog* report" or "radiolog* FINDINGs")).mp. [mp=title, book title, abstract, original title, name of substance word, subject heading word, floating sub-heading word, keyword heading word, organism supplementary concept word, protocol supplementary concept word, rare disease supplementary concept word, unique identifier, synonyms] |
| 21. | (QUALITY adj2 INFORMATION).ti,ab. |
| 22. | "diagnostic information".mp. |
| 23. | (INTERVENTION* adj5 ("IMAGing FINDING*" or "IMAGing RESULTs" or "imaging report" or "MRI REPORT" or "MRI results" or "MRI finding*" or "X-RAY REPORT" or "X-ray results" or "x-ray finding*" or "CT REPORT" or "CT results" or "CT findings" or "RADIOLOG* results" or "radiolog* report" or "radiolog* FINDINGs")).mp. [mp=title, book title, abstract, original title, name of substance word, subject heading word, floating sub-heading word, keyword heading word, organism supplementary concept word, protocol supplementary concept word, rare disease supplementary concept word, unique identifier, synonyms] |
| 24. | biopsychosocial.ab,ti. |
| 25. | exp diagnostic imaging/ or "diagnostic imag*".mp. or exp computer assisted tomography/ or exp nuclear magnetic resonance imaging/ |
| 26. | exp computer assisted tomography/ or "computed tomograph*".mp. or CT.mp. |
| 27. | (ultrasound or ultrasonograph*).mp. [mp=title, book title, abstract, original title, name of substance word, subject heading word, floating sub-heading word, keyword heading word, organism supplementary concept word, protocol supplementary concept word, rare disease supplementary concept word, unique identifier, synonyms] |
| 28. | "x?ray".mp. or exp X ray/ |
| 29. | exp Radiology/ or exp Radiologists/ or RADIOLOG*.mp. or exp Radiology Information Systems/ |
| 30. | "radiograph*".mp. or exp radiography/ |
| 31. | IMAGING.mp. or exp Magnetic Resonance Imaging/ or "magnetic resonance".mp. or MRI.mp. |
| 32. | LUMBAGO.mp. or exp low back pain/ or "low back pain".mp. |
| 33. | BACKACHE.mp. or exp backache/ |
| 34. | exp animal/ |
| 35. | exp human/ |
| 36. | exp musculoskeletal pain/ or musculoskeletal.mp. or musculo?skeletal.mp. |
| 37. | exp Patellofemoral Pain Syndrome/ or exp Back Pain/ or exp Shoulder Pain/ or exp Neck Pain/ or exp foot pain/ |
| 38. | osteoarthriti*.mp. or exp hand osteoarthritis/ or Osteoarthritis, Hip/ or exp Osteoarthritis/ or exp Osteoarthritis, Spine/ or exp Osteoarthritis, Knee/ |
| 39. | spondylosis.mp. or exp Spondylosis/ or exp cervical spondylosis/ |
| 40. | ("foot pain" or "heel pain" or "ankle pain" or "knee pain" or "patellofemoral pain" or "hip pain" or "shoulder pain" or "subacromial pain" or "elbow pain" or "hand pain" or "wrist pain" or radicular or sciatic*).mp. |
| 41. | exp radicular pain/ or exp heel pain/ or exp scapular pain/ or exp arm pain/ or exp spinal pain/ or exp wrist pain/ or exp ankle pain/ or exp discogenic pain/ or exp hand pain/ or exp chronic pain/ |
| 42. | 32 or 33 or 36 or 37 or 38 or 39 or 40 or 41 |
| 43. | 25 or 26 or 27 or 28 or 29 or 30 or 31 |
| 44. | 2 or 3 or 4 or 5 or 6 or 7 or 8 or 9 or 10 or 11 or 12 or 13 or 14 or 15 or 16 or 17 or 18 or 19 or 20 or 21 or 22 or 23 or 24 |
| 45. | 42 and 43 and 44 |
| 46. | 34 and 35 |
| 47. | 34 not 46 |
| 48. | 45 not 47 |
| 49. | 48 not 1 |
|  |  |
|  | **AMED** |
| 1. | Pain/ or exp Musculoskeletal disease/ or Musculoskeletal system/ or musculoskeletal.mp. |
| 2. | "(chronic or persistent) pain".mp. [mp=title, book title, abstract, original title, name of substance word, subject heading word, floating sub-heading word, keyword heading word, organism supplementary concept word, protocol supplementary concept word, rare disease supplementary concept word, unique identifier, synonyms] |
| 3. | exp Low back pain/ or "low back pain".mp. |
| 4. | back pain.mp. or exp Backache/ |
| 5. | spinal.mp. |
| 6. | exp Neck pain/ or "neck pain".mp. |
| 7. | Neuralgia/ |
| 8. | exp Sciatica/ or sciatica.mp. or radicular.mp. [mp=title, book title, abstract, original title, name of substance word, subject heading word, floating sub-heading word, keyword heading word, organism supplementary concept word, protocol supplementary concept word, rare disease supplementary concept word, unique identifier, synonyms] |
| 9. | exp joint disease/ or exp muscular disease/ |
| 10. | Osteoarthritis/ or osteoarthritis.mp. |
| 11. | ("arm pain" or "shoulder pain" or "subacromial pain" or "elbow pain" or "wrist pain" or "hand pain").mp. [mp=title, book title, abstract, original title, name of substance word, subject heading word, floating sub-heading word, keyword heading word, organism supplementary concept word, protocol supplementary concept word, rare disease supplementary concept word, unique identifier, synonyms] |
| 12. | ("leg pain" or "hip pain" or "knee pain" or "patellofemoral pain" or "ankle pain" or "heel pain" or "foot pain").mp. [mp=title, book title, abstract, original title, name of substance word, subject heading word, floating sub-heading word, keyword heading word, organism supplementary concept word, protocol supplementary concept word, rare disease supplementary concept word, unique identifier, synonyms] |
| 13. | (lumbago or backache).mp. |
| 14. | exp Diagnostic imaging/ or imaging.mp. |
| 15. | exp Radiography/ or radiograph*.mp. |
| 16. | "radiolog*".m_titl. |
| 17. | Magnetic resonance imaging/ or "magnetic resonance imaging".mp. |
| 18. | MRI.mp. |
| 19. | Tomography x ray computed/ or computed tomography.mp. |
| 20. | CT.m_titl. |
| 21. | (ultrasound or ultrasonograph*).mp. [mp=title, book title, abstract, original title, name of substance word, subject heading word, floating sub-heading word, keyword heading word, organism supplementary concept word, protocol supplementary concept word, rare disease supplementary concept word, unique identifier, synonyms] |
| 22. | x-ray.mp. |
| 23. | Patient education/ or patient education.mp. |
| 24. | Health education/ or health education.mp. |
| 25. | exp Health Communication/ or "HEALTH COMMUNICATION".mp. |
| 26. | (epidemiolog* adj2 (data or information)).mp. [mp=title, book title, abstract, original title, name of substance word, subject heading word, floating sub-heading word, keyword heading word, organism supplementary concept word, protocol supplementary concept word, rare disease supplementary concept word, unique identifier, synonyms] |
| 27. | (prevalence adj2 (data or information)).mp. [mp=title, book title, abstract, original title, name of substance word, subject heading word, floating sub-heading word, keyword heading word, organism supplementary concept word, protocol supplementary concept word, rare disease supplementary concept word, unique identifier, synonyms] |
| 28. | (pain adj2 education).mp. [mp=title, book title, abstract, original title, name of substance word, subject heading word, floating sub-heading word, keyword heading word, organism supplementary concept word, protocol supplementary concept word, rare disease supplementary concept word, unique identifier, synonyms] |
| 29. | psychoeducation*.mp. [mp=title, book title, abstract, original title, name of substance word, subject heading word, floating sub-heading word, keyword heading word, organism supplementary concept word, protocol supplementary concept word, rare disease supplementary concept word, unique identifier, synonyms] |
| 30. | (communicat* adj3 ("IMAGing FINDINGs" or "IMAGing RESULTs" or "imaging report" or " MRI findings" or "MRI results" or "MRI REPORT*" or "X-RAY findings" or "X-Ray results" or "X-Ray REPORT" or "CT findings" or "CT REPORT" or "CT results" or "RADIOLOG* FINDINGS*" or "radiolog* results" or "radiolog* report")).mp. [mp=title, book title, abstract, original title, name of substance word, subject heading word, floating sub-heading word, keyword heading word, organism supplementary concept word, protocol supplementary concept word, rare disease supplementary concept word, unique identifier, synonyms] |
| 31. | ((WRITTEN or PRINTED or ORAL) adj INFORMATION).mp. [mp=title, book title, abstract, original title, name of substance word, subject heading word, floating sub-heading word, keyword heading word, organism supplementary concept word, protocol supplementary concept word, rare disease supplementary concept word, unique identifier, synonyms] |
| 32. | (interpretation adj3 ("IMAGing FINDINGs" or "IMAGing RESULTs" or "imaging report" or " MRI findings" or "MRI results" or "MRI REPORT*" or "X-RAY findings" or "X-Ray results" or "X-Ray REPORT" or "CT findings" or "CT REPORT" or "CT results" or "RADIOLOG* FINDINGS*" or "radiolog* results" or "radiolog* report")).mp. [mp=title, book title, abstract, original title, name of substance word, subject heading word, floating sub-heading word, keyword heading word, organism supplementary concept word, protocol supplementary concept word, rare disease supplementary concept word, unique identifier, synonyms] |
| 33. | (contextuali* adj3 ("IMAGing FINDINGs" or "IMAGing RESULTs" or "imaging report" or " MRI findings" or "MRI results" or "MRI REPORT*" or "X-RAY findings" or "X-Ray results" or "X-Ray REPORT" or "CT findings" or "CT REPORT" or "CT results" or "RADIOLOG* FINDINGS*" or "radiolog* results" or "radiolog* report")).mp. [mp=title, book title, abstract, original title, name of substance word, subject heading word, floating sub-heading word, keyword heading word, organism supplementary concept word, protocol supplementary concept word, rare disease supplementary concept word, unique identifier, synonyms] |
| 34. | ((perception or perceive) adj3 ("IMAGing FINDINGs" or "IMAGing RESULTs" or "imaging report" or " MRI findings" or "MRI results" or "MRI REPORT*" or "X-RAY findings" or "X-Ray results" or "X-Ray REPORT" or "CT findings" or "CT REPORT" or "CT results" or "RADIOLOG* FINDINGS*" or "radiolog* results" or "radiolog* report")).mp. [mp=title, book title, abstract, original title, name of substance word, subject heading word, floating sub-heading word, keyword heading word, organism supplementary concept word, protocol supplementary concept word, rare disease supplementary concept word, unique identifier, synonyms] |
| 35. | (reassur* adj3 ("IMAGing FINDINGs" or "IMAGing RESULTs" or "imaging report" or " MRI findings" or "MRI results" or "MRI REPORT*" or "X-RAY findings" or "X-Ray results" or "X-Ray REPORT" or "CT findings" or "CT REPORT" or "CT results" or "RADIOLOG* FINDINGS*" or "radiolog* results" or "radiolog* report")).mp. [mp=title, book title, abstract, original title, name of substance word, subject heading word, floating sub-heading word, keyword heading word, organism supplementary concept word, protocol supplementary concept word, rare disease supplementary concept word, unique identifier, synonyms] |
| 36. | biopsychosocial.mp. |
| 37. | "cognitive behavio?ral therapy".mp. |
| 38. | "behavio?r* change".mp. [mp=title, book title, abstract, original title, name of substance word, subject heading word, floating sub-heading word, keyword heading word, organism supplementary concept word, protocol supplementary concept word, rare disease supplementary concept word, unique identifier, synonyms] |
| 39. | (behavio?r adj2 intervention).mp. |
| 40. | exp Animals/ |
| 41. | exp humans/ |
| 42. | exp Arthritis rheumatoid/ |
| 43. | exp Neoplasms/ or neoplasms.mp. or sarcoma*.mp. or osteosarcoma*.mp. or tumo?r*.mp. or cancer.mp. |
| 44. | Central nervous system/ or central nervous system.mp. |
| 45. | Central nervous system disease/ |
| 46. | exp Tooth disease/ or exp Dentistry/ or dentistry.mp. |
| 47. | 1 or 2 or 3 or 4 or 5 or 6 or 7 or 8 or 9 or 10 or 11 or 12 or 13 |
| 48. | 42 or 43 or 44 or 45 or 46 |
| 49. | 40 and 41 |
| 50. | 40 not 49 |
| 51. | 14 or 15 or 16 or 17 or 18 or 19 or 20 or 21 or 22 |
| 52. | 23 or 24 or 25 or 26 or 27 or 28 or 29 or 30 or 31 or 32 or 33 or 34 or 35 or 36 or 37 or 38 or 39 |
| 53. | 47 and 51 and 52 |
| 54. | 53 not 50 |
| 55. | 54 not 48 |
|  |  |
|  | **CINAHL** |
| S40 | S39 NOT S38 |
| S39 | S35 AND S36 AND S37 |
| S38 | S28 OR S29 OR S30 OR S31 OR S32 OR S33 OR S34 |
| S37 | S20 OR S21 OR S22 OR S23 OR S24 OR S25 OR S26 OR S27 |
| S36 | S11 OR S12 OR S13 OR S14 OR S15 OR S16 OR S17 OR S18 OR S19 |
| S35 | S1 OR S2 OR S3 OR S4 OR S5 OR S6 OR S7 OR S8 OR S9 OR S10 |
| S34 | (MH "Tooth Diseases+") |
| S33 | (MH "Dentistry+") |
| S32 | (MH "Central Nervous System+") OR (MH "Central Nervous System Diseases+") |
| S31 | (MH "Rheumatic Diseases+") OR (MH "Nervous System Diseases+") OR (MH "Skin and Connective Tissue Diseases+") |
| S30 | (MH "Neoplasms+") OR AB neoplasm* |
| S29 | (MH "Arthritis, Infectious+") OR (MH "Arthritis, Juvenile Rheumatoid") OR (MH "Arthritis, Rheumatoid+") OR (MH "Arthritis, Psoriatic") OR (MH "Spondylarthritis+") OR (MH "Reiter Disease") |
| S28 | (MH "Animals+") |
| S27 | (AB "report* strateg*") OR (AB "(epidemiolog* OR prevalence) N2 (data or information)") OR (AB "prevalence information") OR (AB "pain N2 education") OR (AB "behavio#r* N2 intervention*") OR (AB "communicat* N2 (result* or finding*)") OR (AB "pain neurobiology") OR (AB "report interpretation N1 (radiolog* OR MRI OR CT OR XR)") OR (AB "diagnostic information") |
| S26 | "contextualis*" |
| S25 | "reassur*" |
| S24 | biopsychosocial |
| S23 | (MM "Models, Biopsychosocial") OR (MH "Models, Psychological+") |
| S22 | (MH "Psychoeducation") OR "psychoeducation*" |
| S21 | (MH "Education, Medical+") OR AB "behavio* change" |
| S20 | (MH "Patient Education+") OR AB "health behavio*" |
| S19 | "X#ray" |
| S18 | "CT" |
| S17 | "MRI" |
| S16 | (MH "Magnetic Resonance Imaging+") OR ""magnetic resonance imaging"" |
| S15 | (MH "Tomography, X-Ray Computed+") OR ""computed tomography"" |
| S14 | "radiolog*" OR (MH "Radiology Information Systems+") OR (MM "Radiology Service") |
| S13 | radiograph* |
| S12 | (MH "Radiography+") OR (MM "Radiography, Computed") OR (MH "Tomography, X-Ray+") |
| S11 | AB "diagnostic imaging" |
| S10 | spondylo* |
| S9 | TI pain |
| S8 | osteoarthrit* |
| S7 | MM osteoarthritis |
| S6 | MW musculoskeletal |
| S5 | musculoskeletal |
| S4 | AB (lumbar or lumbosacral or lumbago or sciatica) |
| S3 | MM back pain+ |
| S2 | MM low back pain |
| S1 | AB (leg or ankle or heel or foot or knee or patellofemoral or hip or back or low back or lumbar or spinal or neck or arm or shoulder or subacromial or elbow or wrist or hand or radicular or sciatic) pain |
|  |  |
|  | **PSYCINFO** |
| 32 | MAINSUBJECT.EXACT.EXPLODE("Roentgenography") OR radiograph* OR x-ray OR CT OR (computed tomography) OR MRI OR imaging OR MAINSUBJECT.EXACT.EXPLODE("Magnetic Resonance Imaging") OR (diagnostic imaging) |
| 31 | tiab(REASSUR*) OR tiab(CONTEXTUALI*) OR tiab(communicat*) OR tiab(INTERPRET*) OR tiab(REPORT*) |
| 30 | (tiab(INTERVENTION* NEAR/5 "IMAGING FINDINGS*" OR "IMAGING RESULTS*" OR "MRI REPORT*" OR "X-RAY" REPORT* " OR " CT REPORT* " OR RADIOGRAPHY* OR " RADIOLOGICAL FINDINGS* "") OR tiab(BEHAVIO* NEAR/2 INTERVENTION*) OR (MAINSUBJECT.EXACT.EXPLODE("Biopsychosocial Approach") OR tiab(biopsychosocial)) OR (MAINSUBJECT.EXACT.EXPLODE("Pain Perception") OR tiab(perception)) OR ((WRITTEN OR PRINTED OR ORAL) NEAR/1 INFORMATION) OR (tiab("PAIN EDUCATION") OR tiab("PAIN NEUROPHYSIOLOGY") OR tiab("PAIN NEUROBIOLOGY")) OR (MAINSUBJECT.EXACT.EXPLODE("Client Education") OR "patient education" OR reassur* OR contextuali*) OR ((EPIDEMIOLOGIC* OR PREVALENCE OR DIAGNOS*) NEAR/2 (DATA OR INFORMATION)) OR (tiab(REASSUR*) OR tiab(CONTEXTUALI*) OR tiab(communicat*) OR tiab(INTERPRET*) AND tiab(REPORT*))) AND ((Musculoskeletal OR ("arm pain" OR "shoulder pain" OR "elbow pain" OR "wrist pain" OR "hand pain") OR MAINSUBJECT.EXACT.EXPLODE("Back Pain") OR MAINSUBJECT.EXACT.EXPLODE("Musculoskeletal Disorders") OR ("neck pain" OR "spinal pain") OR lumbago OR Backache OR osteoarthriti* OR MAINSUBJECT.EXACT.EXPLODE("Arthritis") OR ("leg pain" OR "hip pain" OR "knee pain" OR "ankle pain" OR "heel pain" OR "foot pain") OR (neuralgia OR sciatica OR radicular)) OR "low back pain") AND ((MAINSUBJECT.EXACT.EXPLODE("Roentgenography") OR radiograph*) OR x-ray OR CT OR (computed tomography) OR MRI OR imaging OR MAINSUBJECT.EXACT.EXPLODE("Magnetic Resonance Imaging") OR (diagnostic imaging)) |
| 29 | tiab(INTERVENTION* NEAR/5 "IMAGING FINDINGS*" OR "IMAGING RESULTS*" OR "MRI REPORT*" OR "X-RAY" REPORT* " OR " CT REPORT* " OR RADIOGRAPHY* OR " RADIOLOGICAL FINDINGS* "") OR tiab(BEHAVIO* NEAR/2 INTERVENTION*) OR (MAINSUBJECT.EXACT.EXPLODE("Biopsychosocial Approach") OR tiab(biopsychosocial)) OR (MAINSUBJECT.EXACT.EXPLODE("Pain Perception") OR tiab(perception)) OR ((WRITTEN OR PRINTED OR ORAL) NEAR/1 INFORMATION) OR (tiab("PAIN EDUCATION") OR tiab("PAIN NEUROPHYSIOLOGY") OR tiab("PAIN NEUROBIOLOGY")) OR (MAINSUBJECT.EXACT.EXPLODE("Client Education") OR "patient education" OR reassur* OR contextuali*) OR ((EPIDEMIOLOGIC* OR PREVALENCE OR DIAGNOS*) NEAR/2 (DATA OR INFORMATION)) OR (tiab(REASSUR*) OR tiab(CONTEXTUALI*) OR tiab(communicat*) OR tiab(INTERPRET*) AND tiab(REPORT*)) |
| 28 | (Musculoskeletal OR ("arm pain" OR "shoulder pain" OR "elbow pain" OR "wrist pain" OR "hand pain") OR MAINSUBJECT.EXACT.EXPLODE("Back Pain") OR MAINSUBJECT.EXACT.EXPLODE("Musculoskeletal Disorders") OR ("neck pain" OR "spinal pain") OR lumbago OR Backache OR osteoarthriti* OR MAINSUBJECT.EXACT.EXPLODE("Arthritis") OR ("leg pain" OR "hip pain" OR "knee pain" OR "ankle pain" OR "heel pain" OR "foot pain") OR (neuralgia OR sciatica OR radicular)) OR "low back pain" |
| 27 | "low back pain" |
| 26 | tiab(INTERVENTION* NEAR/5 "IMAGING FINDINGS*" OR "IMAGING RESULTS*" OR "MRI REPORT*" OR "X-RAY" REPORT* " OR " CT REPORT* " OR RADIOGRAPHY* OR " RADIOLOGICAL FINDINGS* "") |
| 25 | tiab(BEHAVIO* NEAR/2 INTERVENTION*) |
| 24 | MAINSUBJECT.EXACT.EXPLODE("Biopsychosocial Approach") OR tiab(biopsychosocial) |
| 23 | MAINSUBJECT.EXACT.EXPLODE("Pain Perception") OR tiab(perception) |
| 22 | ((WRITTEN OR PRINTED OR ORAL) NEAR/1 INFORMATION) |
| 21 | tiab("PAIN EDUCATION") OR tiab("PAIN NEUROPHYSIOLOGY") OR tiab("PAIN NEUROBIOLOGY") |
| 20 | MAINSUBJECT.EXACT.EXPLODE("Client Education") OR "patient education" OR reassur* OR contextuali* |
| 19 | (EPIDEMIOLOGIC* OR PREVALENCE OR DIAGNOS*) NEAR/2 (DATA OR INFORMATION) |
| 18 | x-ray |
| 17 | imaging |
| 16 | MRI |
| 15 | Computed tomography |
| 14 | CT |
| 13 | MAINSUBJECT.EXACT.EXPLODE("Magnetic Resonance Imaging") |
| 12 | Diagnostic imaging |
| 11 | musculoskeletal |
| 10 | MAINSUBJECT.EXACT.EXPLODE("Musculoskeletal Disorders") |
| 9 | MAINSUBJECT.EXACT.EXPLODE("Back Pain") |
| 8 | "arm pain" OR "shoulder pain" OR "elbow pain" OR "wrist pain" OR "hand pain" |
| 7 | "leg pain" OR "hip pain" OR "knee pain" OR "ankle pain" OR "heel pain" OR "foot pain" |
| 6 | MAINSUBJECT.EXACT.EXPLODE("Arthritis") |
| 5 | osteoarthriti* |
| 4 | Backache |
| 3 | lumbago |
| 2 | "neck pain" OR "spinal pain" |
| 1 | neuralgia OR sciatica OR radicular |

Supplementary Table 2 - Study selection template and examples

| **Author** | **Title** | **EK Full text decision** | **AM Full text decision** | **Rejection Reason**  1- not relating to MSK,  2- not targeting imaging communication,  3 - non experimental study,  4 - Not primary Study  5 - Duplicate | **additional notes** | **Full text Final Decision** | **Manual search performed** | **Contact Author required and response** |
| --- | --- | --- | --- | --- | --- | --- | --- | --- |
| Alhowimel, Ahmed; Alotaibi, Mazyad; Coulson, Neil; Radford, Kathryn | Psychosocial consequences of diagnosing nonspecific low-back pain radiologically: a qualitative study | include | include |  |  | include | Complete | not required |
| Asenlöf, Pernilla; Denison, Eva; Lindberg, Per | Individually tailored treatment targeting activity, motor behavior, and cognition reduces pain-related disability: a randomized controlled trial in patients with musculoskeletal pain | exclude | exclude | 2 |  | exclude |  |  |

Supplementary table 3 - Study characteristics

| First Author/  Year | Country/  Setting/  MSK area | Study design/  Numbers (n) | Outcome measures | Summary of results |
| --- | --- | --- | --- | --- |
| Ash  2008 | United States,  Primary care, Low back pain and radiculopathy | RCT. Patients and clinicians blinded and unblinded to Lumbar MRI results/  (n=246) | Roland Morris, Visual Analogue scale, absenteeism, Short Form 36, Self-efficacy, Fear Avoidance Questionnaire, | All Outcomes at 6 weeks were similar for blinded and unblinded groups, except for the general health subscale on the SF-36, Which improved more than for the unblinded group (P=0.08) |
| Bossen  2013 | United States, Secondary care, Hand and upper extremity | Observational Cross sectional study. Participants provided two hypothetical reports one of which was reworded/  (n =100) | Self assessment manikin  Satisfaction, usefulness understanding bespoke likert scales | Questionnaires revealed significantly higher satisfaction, usefulness, understandability, pleasure and lower arousal with reworded reports |
| Fried  2018 | United States, Primary care, Low back pain | Retrospective analysis of electronic records of two groups: one with prevalence statement and one without/  (n=375) | Number of primary care follow-ups, ED visits, frequency/typeof repeat imaging, surgery, physical therapy, medication, | Patients in the statement group were 12% less likely to be referred to a spine specialist (137 of 187 [73%] vs159 of 188 [85%];P= .007) and were 7% less likely to undergo repeat imaging (seven of 187 [4%] vs 20 of 188[11%];P= 0.01). No change in surgery or medication |
| Jarvik  2020 | United States, Primary care, Low back pain | RCT comparing standard lumbar spine imaging reports or reports containing age-appropriate prevalence data (238,886) | spine-related RVUs:  Clinical consultations, opioid prescribing, subsequent imaging, spine injections, other back related medical costs | Small but significant decrease in the likelihood of opioid prescribing from a study clinician within 1 year of the intervention (odds ratio, 0.95; 95% CI, 0.91 to 1.00;P= .04). no difference in RVU or other outcomes |
| Karran  2018 | Australia, Spinal outpatient  clinic at a metropolitan hospital, Low back pain | Feasibility study  (31) | Fulfilment of study feasibility criteria alongside Pain disability and kinesiophobia | N/A |
| Karran  2017 | Australia, virtual patients, Low back pain | RCT. Group 1: imaging and pre-information about normal findings. Group 2: imaging only. Group 3: quality information without imaging. Group 1 divided to receive either a standard report, or enhanced report containing altered terminology and epidemiological information)  (n=660) | Back-related perceptions  (BRP), a composite score derived from three numeric rating scale scores exploring perceptions of spinal condition, recovery concerns and planned activity. The secondary outcomes were satisfaction and kinesiophobia | Best practice care resulted in more positive BRPs than receiving imaging results. Receiving prior information about normal findings had no impact. Enhanced reporting strategies significantly improved BRPs (F(1,275)=13.06,p<0.001,η2p=.05). satisfaction (F(2,553)=7.5,p=0.001,η2p=.03) and kinesiophobia (F(2,553)=3.0,p=0.050,η2p=.01). |
| Medalian  2019 | Australia, Online simulated scenarios, Low back pain | Online RCT.  Participants presented with a “virtual patient” via online survey and randomly allocated to standard imaging report or a standard report with additional epidemiological information  (n=247) | Composite Back Related Perceptions questionnaire  Health satisfaction  Tampa Scale Kinesiophobia | There was a small effect of group on BRP [F(12,444)=6.75,p=0.010] with participants who received the additional epidemiological information demonstrating more positive perceptions |
| Rajasekaran  2021 | India, Primary care,  Low Back pain | RCT  Phase I - patients were randomized to a factual explanation of their MRI report or reassured that the MRI findings showed normal changes.  (n=44) | Phase I - baseline and 6 weeks  The severity of pain (visual analogue scale),  The perception of their status of spine and disease (Pain Self Efficacy Questionnaire, Short form Survey-SF-12) | Factual group had a more negative perception of their spinal condition, increased catastrophisation, decreased pain improvement, and poorer functional status  (p<0.001) all outcomes. |
| Stanton  2020 | Australia, Primary care, Knee OA | feasibility study.  Participants randomised into the Pain Science Education group or Control group, each receiving 4 in-person weekly treatments, then 4 weeks of at-home activities (weekly telephone check-in)  (n=20) | Feasibility and acceptability outcomes,  Pain Visual Analogue Scale, Western Ontario Mcmaster Universities OA Index (WOMAC),  Pain Self Efficacy Questionnaire,  Brief Fear of Movement scale for OA, Pain Catastrophising Scale, Pain Beliefs Questionnaire, Revised Neurophysiology of Pain Questionnaire | N/A |
| Weeks  2020 | United States, Primary and Secondary care, Low back pain | Retrospective before after analysis of patients receiving epidemiological data in reports and those without  (n=6904) | expenditures for four clinician visit types (primary care, chiropractic, physical therapy, and specialty care), three testing modalities (nerve conduction testing, MRI, and non-MRI), and five treatment types (spinal facet injections, opioid and muscle relaxant prescriptions, fusion spine surgery, and non-fusion spine surgery). | Relative to the control group, total spine-related expenditures in the intervention group fell by approximately $332 per member per year. |
| Zhuang  2019 | China, Spinal surgical unit, Low back pain | 3-Arm RCT  (45) | Questionnaire consisted of 20 multiple choice questions to evaluate four components of related knowledge: (a) lumbar anatomy, (b) physiology (c) their disease (d) surgical plan (Appendix A). Questionnaire surveyed the patients’ satisfaction with the anatomy, physiology, my disease, surgery, and overall components of their consultation using a 0–10 Likert rating scale | Patients educated with personalised 3D printed models demonstrated an expanded level of understanding than patients educated with CT & MRI imaging (care-as-usual) (P<0.05) and 3D reconstructions (P<0.05). Personalized 3D printed models also resulted in a higher degree of patient satisfaction (P<0.05) |

Supplementary Figure 2 – Forest plots of pain, disability and fear in short (<6wks), medium (3-6 Months) and long term (>1yr)


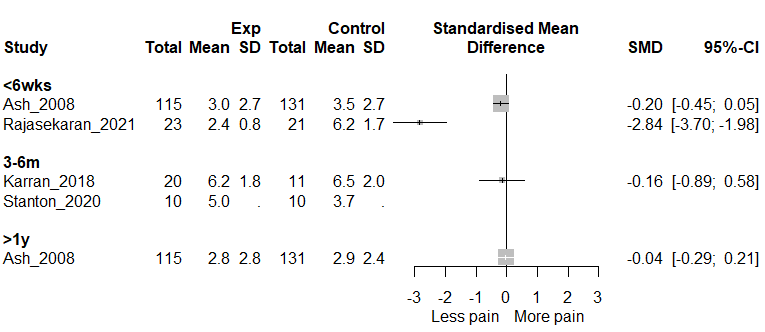


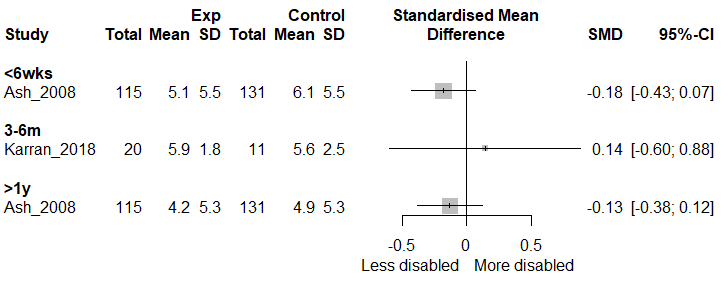


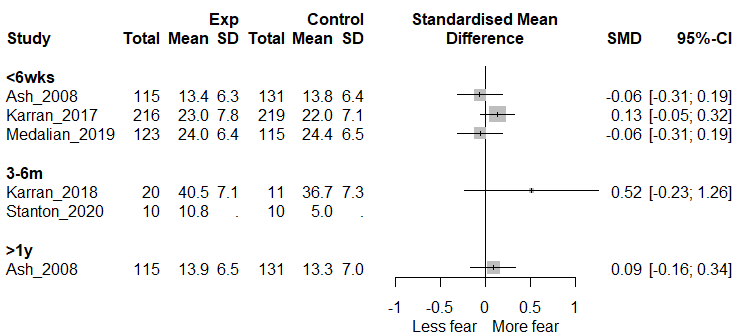


Supplementary Table 4. Intervention description (TIDieR)

|  | Authors | Ash LM, Modic MT, Obuchowski NA, Ross JS, Grooff PN |
| --- | --- | --- |
| 1 | Brief Name | Effects of diagnostic information on outcomes in LBP |
| 2 | Study Methodology | Quantitative – prospective randomised controlled trial |
| 3 | Why (include explicit rationale, theory or goal of the elements essential to the intervention)  Coherence of target/intervention/outcome  Intervention design process | - Author prospectively proposes that knowledge of MRI results affect outcomes (pain related disability, pain intensity, general wellbeing, absenteeism, Self-efficacy and Fear Avoidance) via reassurance and reduced anxiety. - Unclear target for intervention - No established theoretical framework or construct identified - No measurement of reassurance or anxiety - No intervention design process |
|  |  | Painter criteria: no theory |
| 4 | What  Include details of design process  intervention materials and procedures and intended target of intervention | All participants receive assessment and treatment as normal. Blinded group did not receive MRI results and unblinded group received results. Treatment continued as per initial plan. Patients and treating clinician allocated to receiving results or not receiving results group after clinical examination by study team. MRI scan (T1 axial and sagittal and T2 sagittal and axial fast spin-echo images) performed at presentation and at 6 weeks. |
| 5 | Who provided intervention (inc expertise/background) | Initial clinical examination performed by treating physician. Images of patients in receiving results arm interpreted by on duty radiologist (one of eight, two of whom involved in the study). Images of blinded patients reviewed by study author for serious pathology.  3 independent radiologists (15-, 20- and 10-years’ experience) recorded |
| 6 | Where | Centre for the spine, primary care units and regional satellites and the emergency department for the Cleveland Clinic Foundation |
| 7 | When and how much (was the intervention delivered) | Withholding of the results occurred for 6 months while treatment as usual continued |
| 8 | Tailoring and modifications | Nil |
| 9 | How well (adherence and fidelity planning and execution) | 20% lost to follow up |
|  | Outcomes | - Roland pain related function questionnaire - Visual pain analogue scale - Short form 36 - Self-efficacy - Fear Avoidance Questionnaire |
|  | Authors | Bossen J, Hageman M, King J, Ring, D |
| 1 | Brief Name | Does rewording MRI reports improve understanding |
| 2 | Study Methodology | Quantitative – observational cross-sectional |
| 3 | Why (include explicit rationale, theory or goal of the elements essential to the intervention)  Coherence of target/intervention/outcome | - Author proposes that limited understanding and misinterpretation of imaging findings may affect health and satisfaction outcomes and that rewording of MRI reports may result in better patient understanding - No established theoretical framework or construct identified. |
|  |  | Painter criteria: no theory |
| 4 | What  Include details of design process  intervention materials and procedures and intended target of intervention | Original reports (not relating the participants) were reworded to below eight grade reading level using MS Word. Neutral descriptive words, analogies, optimistic interpretation chosen.  Both the original and reworded report was presented to the patient |
| 5 | Who provided (inc expertise/background) | Study team performed rewording |
| 6 | Where | Orthopaedic hand and upper extremity clinic Boston Massachusetts |
| 7 | When and how much (was the intervention delivered) | Singular simulation |
| 8 | Tailoring and modifications | Nil |
| 9 | How well (adherence and fidelity planning and execution) | 100% follow up, 100% completion |
|  | Outcomes | - Self-assessment manikin - Patient satisfaction - Usefulness and understanding |
|  | Authors | Fried J, Andrew A, Ring N, Pastel D |
| 1 | Brief Name | Changes in healthcare utilisation after inclusion of epidemiological data in Lumbar MRI reports |
| 2 | Study Methodology | Quantitative - Longitudinal |
| 3 | Why (include explicit rationale, theory or goal of the elements essential to the intervention)  Include coherence of target, intervention, outcomes | - Author proposes that inclusion of an epidemiological statement influences downstream costs. - No established theoretical framework or construct identified - Unclear target – assumed to be patient via primary care clinician explanation |
|  |  | Painter Criteria: No theory |
| 4 | What  Include details of design process  intervention materials and procedures and intended target of intervention | Addition of comment:  “The following findings are so common in people without low back pain that while we report their presence, they must be interpreted with caution and in the context of the clinical situation.”  Addition of Findings statement:  (prevalence in patients without low back pain), Disk degeneration (decreased T2 signal, height loss, bulge) (91%), Disk T2-signalloss (83%), Disk height loss (56%),Disk bulge (64%), Disk protrusion(32%), Annular tear (38%) |
| 5 | Who provided (inc expertise/background) | Neuroradiologist with 8 yrs experience |
| 6 | Where | Single tertiary academic centre in the US |
| 7 | When and how much (was the intervention delivered) | Single epidemiological statement at point of MRI reporting |
| 8 | Tailoring and modifications | Nil |
| 9 | How well (adherence and fidelity planning and execution) | High exclusion level  Good fidelity |
| 10 | Outcomes | - Frequency and type of repeat imaging - Narcotic prescriptions – continuation, new, change prescription. Total narcotic burden (milligrams times morphine equivalence - Number of primary care follow ups - Number of emergency department visits - Referral to a spine specialist - Number of visits to a spine specialist - Physical therapy referral - Imaging based interventions (epidural, nerve block) - Spinal surgery |
|  | Authors | Jarvik JG, Meier EN, James KT, Gold LS, Tan KW Kessler LG, Suri P, Kallmes DF, Cherkin DC, Deyo RA, Sherman KJ, Halabi SS, Comstock BA, Luetmer PH, Avins AL, Rundell SD, Griffith B, Friedly JL, Lavallee DC, Stephens KA, Turner JA, Bresnahan BW, Heagerty PJ |
| 1 | Brief Name | The effect of Including Prevalence data of spine imaging findings on health care utilisation |
| 2 | Study Methodology | Randomised controlled trial |
| 3 | Why (include explicit rationale, theory or goal of the elements essential to the intervention)  Include coherence of target, intervention, outcomes | - Including prevalence data of degenerative findings in normal population to imaging reports will reduce healthcare utilisation and opioid prescription. - No rationale/model explaining how this might occur - No established theoretical framework or construct identified - Unclear if target is patient or reporting clinician |
|  |  | Painter Criteria: No theory |
| 4 | What  Include details of design process  intervention materials and procedures and intended target of intervention | Including benchmarked age-appropriate and imaging modality appropriate prevalence data  Unclear target for intervention whether patient or reporting clinician |
| 5 | Who provided (inc expertise/background) | Study radiologist using electronic reporting system |
| 6 | Where | For profit hospital in the US.  4 integrated health care systems: Kaiser Permanente Northern California; Henry Ford Health System in Michigan; Kaiser Permanente Washington; and Mayo Clinic Health System in Minnesota and Wisconsin. |
| 7 | When and how much (was the intervention delivered) | 2013-2016. 238,886 patients had benchmark prevalence data included in their reports |
| 8 | Tailoring and modifications | Age and modality specific modifications to prevalence data |
| 9 | How well (adherence and fidelity planning and execution) | Small change of opioid outcome measure compared to protocol |
| 10 | Outcomes | Primary outcome: Spine related relative value units  secondary outcomes: (1)  total morphine equivalent dose (MED) prescribed per patient (2) cumulative spine-related total RVUs 2years after index imaging; (3) subsequent advanced imaging (i.e., number of magnetic resonance imaging [MRI] or computed tomography [CT] studies) within 90 days and 12 months after index imaging study; (4) spine injections and spine surgeries; and (5) other back-related medical costs during 2 years Opioid prescription |
|  | Authors | Karran E, Hillier S, Yau Y, McAuley J, Mosely G |
| 1 | Brief Name | An educational image interpretation intervention for low back pain (GLITtER) |
| 2 | Study Methodology | Quantitative, Quasi RCT - Feasibility study |
| 3 | Why (include explicit rationale, theory or goal of the elements essential to the intervention)  Include coherence of target, intervention, outcomes | - Development of the intervention involved consideration of contemporary conceptual change theory describing a process in which old, previously learned information is replaced by a revised construction of knowledge. This change process requires appreciation of the prior beliefs and understanding of the ‘learner’ and their motivation to engage with new information - Intervention target was to influence a patients’ understanding of their condition such that activity and exercise were considered safe and necessary for optimal recovery |
|  |  | - Painter Criteria: Testing theory - Theoretical framework specified; more than half of the theoretical constructs in intervention or descriptive/explanatory research were measured and explicitly tested. |
| 4 | What  Include details of design process  intervention materials and procedures and intended | Clinicians were trained for 2-2.5 hrs then delivered an intervention involving  Materials:  Visual aid A (used during intervention delivery): graph of prevalence of degenerative features in asymptomatic adults.  Take-home information resource. This was designed as a series of 4 posters to be displayed one week at a time.  Links to online information (incorporated into take-home resource and delivered via smartphone text messages):◦   - The truth about back pain (<https://www.youtube.com/watch?v=b-cBtPSf0Hc>) - Tame the Beast (<https://www.youtube.com/watch?v=ikUzvSph7Z4>) - How to start exercising and stick to it (<https://www.helpguide.org/articles/healthy-living/how-to-start-exercising-and-stick-to-it.htm>) - Understanding pain in less than 5 min   Procedures:  All procedures implemented in the standard consultation were included in the GLITtER consultation. Additional procedures (unique to the GLITtER consultation):   1. Provide detailed information about ‘normal’, age-relevant imaging findings and involve visual aid A. 2. In addition to explaining patient’s imaging findings, ex-plain that: Scans (on their own) do not explain much about:- Your current pain (e.g., why you have good days and bad).- The activity you are capable of, or- How likely you are to recover (because the changes on your scans will still be there when your pain goes away) 3. Re-interpret imaging findings, highlighting ‘positive’ features. E.g.- Demonstrate spinal features that offer structural stability and emphasise the inherent strength of the spine.- Demonstrate musculature and joints–structures that need movement to be optimally healthy 4. Promote using the ‘TICK list’ as a strategy for increasing planned activity/exercise 5. Introduce patient to take-home information 6. Request patient completion of GLITtER checklist and discuss further if required. 7. Text message follow-up: 4×(brief) weekly SMS messages prompted participants to display/read the relevant poster, and provided an active link to the online information recommended on the poster |
| 5 | Who provided (inc expertise/background) | Two study trained physiotherapists with 14- and 13-years’ experience with postgraduate masters degrees |
| 6 | Where | large metropolitan hospital in South Australia |
| 7 | When and how much (was the intervention delivered) | Single session, integrated into a standard SAC consultation. Approximately 10 min’s duration (in addition to standard consultation |
| 8 | Tailoring and modifications | centred standard consultation (as noted). Imaging interpretation tailored according to imaging findings. Exercise advice tailored according to the patient’s age, physical condition, and practical considerations. |
| 9 | How well (adherence and fidelity planning and execution) | Not assessed |
| 10 | Outcomes | - Fulfilment of study feasibility criteria - Pain - disability - Kinesiophobia |
|  | Authors | Karran, Medalian, Hillier, Moseley |
| 1 | Brief Name | Online reporting strategies investigation |
| 2 | Study Methodology | Quantitative, simulated RCT |
| 3 | Why (include explicit rationale, theory or goal of the elements essential to the intervention)  Include coherence of target, intervention, outcomes | - Prior information changes perception - Information is better than non-contextualised image reports - No established theoretical framework or construct identified |
|  |  | Painter Criteria: No theory |
| 4 | What  Include details of design process  intervention materials and procedures and intended | Simulated 3 arm randomised online experiment.  Adult volunteers were provided with a scenario describing a person who injures their back while lifting. Participants were asked to imagine that they were person described in the scenario as they completed the baseline primary outcome measures. The scenario was developed to describe the person’s concern about their slow recovery (three weeks later) and their decision to go to see their GP. Participants were randomly allocated to three groups.  Group 1 received imaging and was pre-informed about normal findings.  Group 2 received imaging (without pre-information).  Group 3 received best practice care: quality information without imaging.  Group 1 was further divided to receive either a standard report, or an ‘enhanced’ report (containing altered terminology and epidemiological information). |
| 5 | Who provided (inc expertise/background) | Online simulations provided by study team |
| 6 | Where | Online |
| 7 | When and how much (was the intervention delivered) | Singular simulation |
| 8 | Tailoring and modifications | Age matched simulations |
| 9 | How well (adherence and fidelity planning and execution) | A total of 788 participants commenced the online study between February and June, 2017. 660 participants completed baseline and post-intervention BRP scores and were included in the analysis |
| 10 | Outcomes | - a composite score derived from three numeric rating scale (NRS) responses. - Kinesiophobia (TSK) - Patient satisfaction rating |
|  | Authors | Medalian Y, Moseley L, Karran E |
| 1 | Brief Name | Online investigation of the impact of adding epidemiological information to imaging reports |
| 2 | Study Methodology | Quantitative Simulated RCT |
| 3 | Why (include explicit rationale, theory or goal of the elements essential to the intervention)  Include coherence of target, intervention, outcomes | - Including epidemiological information (i.e. data summarising the prevalence of degenerative findings in asymptomatic individuals) in imaging reports has been recommended to address the potential for misinterpretation of the relevance of identified degenerative changes - Target: patients and their back-related perceptions - No established theoretical framework or construct identified |
|  |  | Painter Criteria: No theory |
| 4 | What  Include details of design process  intervention materials and procedures and intended | a two-armed randomised controlled online experiment. Non back pain suffering participants were presented with virtual patient scenario and asked to imagine they were this person. Participants were randomly allocated to a situation in which the virtual patient received a standard report or a report that included additional epidemiological information. Participants subsequently completed the primary and secondary outcome measure questions. |
| 5 | Who provided (inc expertise/background) | Participants accessed the study via web-based survey software (https:// www.surveymonkey.com). |
| 6 | Where | online |
| 7 | When and how much (was the intervention delivered) | Singular scenario and outcomes |
| 8 | Tailoring and modifications | Nil |
| 9 | How well (adherence and fidelity planning and execution) | Due to the nature of the study (singular online survey) there are no fidelity issues |
| 10 | Outcomes | Composite Back Related Perceptions questionnaire  Health satisfaction  Tampa Scale Kinesiophobia |
|  | Authors | Rajasekaran S, Raja S, Pushpa B, Ananda K, Prasad S, Rishi M |
| 1 | Brief Name | Testing then Development of adapted MRI report |
| 2 | Study Methodology | Phase I - RCT - evaluation of factual reporting vs reassurance on patients  Phase II – development of terminology for clinical report  Phase III – evaluation of clinical report terminology on HCP’s |
| 3 | Why (include explicit rationale, theory or goal of the elements essential to the intervention)  Include coherence of target, intervention, outcomes | Rationale:   - Authors imply that reassurance may mitigate nocebo effect of MRI findings (phase I) - Language can affect cause fear and catastrophisation (phase II and III) - Target: Phase I - patient, Phase III – clinician - No established theoretical framework or construct identified |
|  |  | Painter Criteria: No Theory |
| 4 | What  Include details of design process  intervention materials and procedures and intended | Phase I  Control group had a full factual explanation of the pathologies reported in their MRI, Intervention group were reassured that their MRI was completely normal with only incidental and age-related findings  Phase-II  a google search was performed to secure the online information available to patients on terminologies frequently used in MRI, and those causing concern and anxiety to patients were identified. An alternate method of ‘clinical reporting’ was evolved, avoiding these terminologies without losing scientific clarity. Modified Pfirrmann grading to substitute disc degeneration, dehydration, desiccation and bulge; Schizas grading for lumbar stenosis; high-intensity zone (HIZ) for annular tears and fissures; ‘close proximity without compression’ to indicate nerve root indentation/impingement or abutment were employed to eliminate terminologies causing fear  Phase III – A clinician stakeholder group went through 20 factual reports and the adapted versions in a blinded fashion and opined on three factors for each report—(1) their assessment of severity of the spinal condition (scale 0−10); (2) their choice of treatment between conservative therapy, injection and surgery; and (3) the probability of requiring surgery (scale 0−10). |
| 5 | Who provided (inc expertise/background) | Phase I – unclear  Phase II – study team  Phase III - 40 health professionals ten each of spine surgeons (SS), general orthopaedic surgeons (OS), orthopaedic residents (OR), and physiotherapists (PT) involved in spine care. |
| 6 | Where | India – exact location not stated |
| 7 | When and how much (was the intervention delivered) | Phase I – singular explanation |
| 8 | Tailoring and modifications | nil |
| 9 | How well (adherence and fidelity planning and execution) | No formal assessment |
| 10 | Outcomes | Phase I - baseline and 6 weeks   - The severity of pain (visual analogue scale—VAS), - The perception of their status of spine and disease (Pain Self Efficacy Questionnaire-PSEQ-2) - Functional status (Short form Survey-SF-12   Phase III   - assessment of severity of the spinal pathology; - choice of treatment between conservative, injections and surgery; - the perceived probability of requiring surgery |
|  | Authors | Stanton T, Karran E, Butler D, Hull M, Schwetlik, Braithwaite F, Jones H, Moseley GL, Hill C, Tomkins-Lane C, Maher C, Bennell K |
| 1 | Brief Name | an RCT investigating the effect of adding Pain Science Education (vs adding sham ultrasound) to an individualised, physiotherapist-led general education and walking program for people with painful knee OA. |
| 2 | Study Methodology | RCT Feasibility study |
| 3 | Why (include explicit rationale, theory or goal of the elements essential to the intervention)  Include coherence of target, intervention, outcomes | - Rationale: Pain science Education shifts conceptualisation of pain from tissue damage to a marker of perceived need to protect the body. This belief can help patients engage with evidence-based treatment such as exercise. - Target: participant but intervention includes training clinicians. - Testing theory - Theoretical framework specified; more than half of the theoretical constructs in intervention or descriptive/explanatory research were measured and explicitly tested. |
|  |  | Painter Criteria: Testing Theory |
| 4 | What  Include details of design process  intervention materials and procedures and intended | **Treating clinician**  The clinician providing PSE received 20 hours of in-depth training from PSE Expert. The clinician providing the Control treatment received 8 hours of training on educational content and sham ultrasound provision. Both therapists received 1 hour of training for the walking program.  **Control Group**  guideline-based general OA education based on Arthritis Australia handbook and physical activity education in addition to an individualised walking program. Including one to one session (weeks 1 to 4) and at home sessions (weeks 5-8)  PLUS, sham ultrasound  Imaging specific content :  “Your doctor may refer you for an x-ray of your knee (or another type of scan) to assist with the diagnosis of osteoarthritis”  **Intervention group**  Pain science education related to OA knee in addition to an individualised walking program. Including one to one sessions (weeks 1 to 4) and at home sessions (weeks 5-8)  Imaging specific content: One to One sessions  X-ray interpretation -  The aim is to ‘dethreaten’ radiological findings. A detailed analysis of participants’ own x-ray was undertaken, focusing on positive features (e.g., excellent bone density) using standardised wording. Education about the poor correlation between x-ray findings and pain was provided  “The severity of changes shown on a knee x-ray do not have much relationship with how much pain you currently have or are likely to have in the future. Pain is complex and influenced by many things – not just what’s going on in your knee”  Imaging specific session - Radiographic changes usually do not relate to pain or to prognosis & What else is going on in the tissues and how does it relate to pain?  Content  • Identifiable changes on x-ray are not pain and many people without  pain have these changes too  • Amazing pain stories  • Pain is an unreliable indicator of tissue damage  • We don’t treat x-rays or scans  • When are scans important?  • ‘Phew, it’s only arthritis! - We will offer an alternative (more  complete) explanation for your OA pain. (- promise!)  Materials:  EP p. 12-15 (Amazing pain stories)  • Stages of OA and % without pain (stats/graph)  • Positive scans in pain-free people (x-rays)  • News story of the runner with ‘terrible’ knees  • Scans are often important in the case of suspected # (following a fall) or suspicion  of serious pathology (rare)  • EPS Nugget 16 p.177 (We stop feeling it way before we stop healing it)  • EPS Nugget 67 p. 194 (We grow like trees)  • *If participants bring up MRI findings, have resources/explanation ready |
| 5 | Who provided (inc expertise/background) | PSE  10years of clinical experience, attended the Noi group Explain Pain course (www.noigroup.com/), and received;20 hours of in-depth training from PSE Expert.  Control treatment  3 years of clinical experience and received;8 hours of training on educational content and sham ultrasound provision. Both therapists received 1 hour of training for the walking program |
| 6 | Where | University of South Australia |
| 7 | When and how much (was the intervention delivered) | X4 weekly 60-90 min one to one sessions  X4 weekly phone calls 15-20 mins with physio |
| 8 | Tailoring and modifications | Individualised content based on PSE paradigm |
| 9 | How well (adherence and fidelity planning and execution) | Thorough adherence and fidelity measures as feasibility study |
| 10 | Outcomes | - Feasibility and acceptability outcomes   AND   - Pain Visual Analogue Scale - Western Ontario McMaster Universities OA Index (WOMAC) - Pain Self Efficacy Questionnaire - Brief Fear of Movement scale for OA - Pain Catastrophising Scale - Pain Beliefs Questionnaire - Revised Neurophysiology of Pain Questionnaire |
|  | Authors | Weeks W, Pike J, Schaeffer C, Devine M, Ventura J, Donath J, Justice B |
| 1 | Brief Name | Epidemiological information in LBP MRI reports reduces costs |
| 2 | Study Methodology | Retrospective before after controlled design |
| 3 | Why (include explicit rationale, theory or goal of the elements essential to the intervention)  Include coherence of target, intervention, outcomes | - Author implies that Including epidemiological data may reduce specialist referral - Target: unclear - No established theoretical framework or construct identified |
| 4 | What  Include details of design process  intervention materials and procedures and intended | Inclusion of epidemiological data adapted from Brinjikji et al (2015) within MRI reports |
|  |  | Painter Criteria: No theory |
| 5 | Who provided (inc expertise/background) | Unclear  Radiology reports amended by radiologist, no details of how this information was conveyed to patients |
| 6 | Where | US - 6 counties surrounding Rochester, New York. |
| 7 | When and how much (was the intervention delivered) | Singular addition of epidemiological data to MRI reports beginning July 1^st^ 2017 |
| 8 | Tailoring and modifications | Nil |
| 9 | How well (adherence and fidelity planning and execution) | N/A as retrospective before after design |
| 10 | Outcomes | per capita utilization rates and PMPM expenditures for four clinician visit types (primary care, chiropractic, physical therapy, and specialty care), three testing modalities (nerve conduction testing, MRI, and non-MRI), and five treatment types (spinal facet injections, opioid and muscle relaxant prescriptions, fusion spine surgery, and non-fusion spine surgery). |
|  | Authors | Zhuang Y, Zhou M, Liu S, Wu J, Wang R, Chen C |
| 1 | Brief Name | Satisfaction of the explanation of back pain using 3D printed models |
| 2 | Study Methodology | Randomised controlled Pilot study |
| 3 | Why (include explicit rationale, theory or goal of the elements essential to the intervention)  Include coherence of target, intervention, outcomes | - Personalised models help patients to understand their condition - Target: patients - No established theoretical framework or construct identified |
| 4 | What  Include details of design process  intervention materials and procedures and intended | 3D printing model was created on an OBJET500 Connex3 system (Stratasys, Eden Prairie, MN,USA) based on CT scans with the assistance of a 3D printing manufacturer (Lan Tian Yang, Fujian, China)  Different education methods were introduced during a face-to-face consultation in half an hour with the surgeon according to the randomly assigned education method (CT & MRI imaging (care-as-usual), 3D volumetric rendering or personalized 3D printing)  One day after patient education session were complete, two questionnaires were administered to prospectively evaluate the level of patients’ pre-operative understanding and satisfaction |
|  |  | Painter Criteria: No Theory |
| 5 | Who provided (inc expertise/background) | Education provided by spinal surgeon |
| 6 | Where | Single centre Fujian Union Hospital May 2017 – June 2018 |
| 7 | When and how much (was the intervention delivered) | Singular education session |
| 8 | Tailoring and modifications | Personalised description of pathology |
| 9 | How well (adherence and fidelity planning and execution) | Not assesses |
| 10 | Outcomes | Questionnaire consisted of 20 multiple choice questions to evaluate four components of related knowledge: (a) lumbar anatomy, (b) physiology (c) their disease (d) surgical plan (Appendix A). Questionnaire surveyed the patients’ satisfaction with the anatomy, physiology, my disease, surgery, and overall components of their consultation using a 0–10 Likert rating scale |

Supplementary Table 5 – Guidance for the coding of BCT’s according to the BCTTv1

| **TRAINING OF DELIVERER OF INTERVENTION**  If education is delivered to a person to subsequently delivering the intervention to patients, then code separately and in addition to direct researcher to patient interventions. In this situation consider:  **Training**  If the study outlines clinician training involving the communication of imaging findings  Code as:   - 4.1 *- instruction on how to perform a behaviour*   Only code as   - 6.1 *demonstration of the behaviour* and 8.1 *behavioural practice/rehearsal* if the intervention explicitly states demonstration.   If training involves provision of information about the effects of the training or not performing the training e.g. “The consequence of performing this behaviour (harmful reporting) is increasing fear and downstream costs”, then code as:   - 5.1 - *information about health consequences*   For retrospective information code as:   - 2.7 - feedback *on outcomes of behaviour*   **Education materials**  If patient education materials are used to train intervention deliverers   - 12.5 *adding objects to environment*   Consider adding additional codes explaining nature of information   - 5.1 *information about health consequences* - 7.*1 prompt/cue* (poster) - 9.2 *pro and cons* - *9.1 Credible source*     If the research team indicates that the patient education material e.g. booklet was intended to act as a reminder to the clinician then also code as   - 7.1 *Prompt cue*, otherwise only code as 12.5. |
| --- |
| **ADDING EPIDEMIOLOGICAL INFORMATION**  Code as patient and clinician target unless explicitly no clinician input to reporting pathway  If adding epidemiological information   - 12.5 – adding objects to the environment - 13.2 - Framing/reframing - 13.3 - Incompatible beliefs |
| **WITHHOLDING IMAGING FINDINGS**  Code as patient and clinician target unless explicitly no clinician input to reporting pathway.  If withholding results   - 12.2 avoidance/reducing exposure for the behaviour |
| **REASSURANCE**  Code as:   - 13.2 – framing/reframing   If the reassurance involves discussion about consequences of a normal age-related findings e.g. The robustness of the spine to increase activity, then code as   - 5.1 – information about health consequences   If the reassurance involves reducing exposure to the MRI report, code as   - 12.2 avoidance/reducing exposure for the behaviour |

Supplementary table 6. The BCTs identified in each study.

| Study author and year | Intervention description | BCT | | | | | | | | | | | |
| --- | --- | --- | --- | --- | --- | --- | --- | --- | --- | --- | --- | --- | --- |
|  |  | Action Planning | Instruction on how to perform behaviour | Information about health consequences | Demonstration of the behaviour | Prompts/cues | Behavioural practice/Rehearsal | Credible source | Avoidance/reducing exposure to cues for the behaviour | Adding objects to the environment | Framing/Reframing | Incompatible beliefs | Number of BCTs per study |
| Ash  (2008) [17] | Patient and clinicians blinded to MRI results |  |  |  |  |  |  |  | x |  |  |  | 1 |
| Bossen  (2013) [61] | MRI reports reworded |  |  |  |  |  |  |  | x |  | x |  | 2 |
| Fried  (2018) [54] | Addition of prevalence statement |  |  |  |  |  |  |  |  | x | x | x | 3 |
| Jarvik  (2020) [55] | Addition of prevalence statement |  |  |  |  |  |  |  |  | x | x | x | 3 |
| Karran  (2018) [58]  GLITtER | Multiple clinician and patient interventions | x | x | x | x | x |  | x |  | x | x | x | 10 |
|  |  |  | x | x | x | x | x |  |  | x |  |  |  |
| Karran  (2017) [48] | Multiple patient interventions |  |  | x |  |  |  |  |  | x | x | x | 4 |
| Medalian  (2019) [56] | Addition of age specific prevalence information |  |  |  |  |  |  |  |  | x | x | x | 3 |
| Rajasekaran  (2021) Phase I [60] | Patient Reassurance of normal findings |  |  |  |  |  |  |  | x |  | x |  | 2 |
| Rajasekaran  (2021) Phase III [60] | Clinician reporting avoiding concerning terminologies |  |  |  |  |  |  |  | x |  | x |  |  |
| Stanton  (2020) [59] | Multiple interventions targeting Clinician and patient | x | x | x | x | x |  |  |  | x | x | x | 9 |
|  |  |  | x | x | x | x | x |  |  | x |  |  |  |
| Weeks  (2020) [57] | Addition of prevalence statement |  |  |  |  |  |  |  |  | x | x | x | 3 |
| Zhuang  (2019) | Use of 3D printed model to explain condition |  |  |  |  |  |  |  |  | x |  |  | 1 |

Supplementary table 7. BCTs and their COM-B targets.

| BCT | Study author/year | Intervention description | Capability | | | | Opportunity | | | | Motivation | | | |
| --- | --- | --- | --- | --- | --- | --- | --- | --- | --- | --- | --- | --- | --- | --- |
|  |  |  | Physical | | Psychol | | Social | | Physical | | Reflective | | Automatic | |
|  |  |  | Skills | Knowledge | Memory attention decision processes | Behavioural regulation | Social influences | Environmental content & resources | Beliefs about capabilities | Beliefs about consequences | Social professional role and identity | optimism | goals | Emotions |
| Action Planning | Karran (2018) [58] | Patient completes exercise plan as part of take-home information |  |  |  | * |  |  |  |  |  |  |  |  |
| Instruction on how to perform a behaviour | Karran (2018) [58] | instruction on imaging interpretation framework |  | ** |  |  |  |  |  |  |  |  |  |  |
|  | Stanton (2020) [59] | 20 hours of in-depth training from Pain Science Education Expert |  | ** |  |  |  |  |  |  |  |  |  |  |
|  | Karran  (2017) [48] | Advice on how to improve activity within best practice care arm |  | ** |  |  |  |  |  |  |  |  |  |  |
| Information about health consequences | Karran (2018) [58] | Explanation that joints need movement to be optimally healthy |  | * |  |  |  |  |  | * |  |  |  |  |
|  | Stanton (2020) [59] | Education that changes on x-ray are not related to pain |  | * |  |  |  |  |  | * |  |  |  |  |
|  | Karran  (2017) [48] | Provision of information that the scan findings do not represent serious damage and movement is recommended |  | * |  |  |  |  |  | * |  |  |  |  |
| Demonstration of the behaviour | Karran et al (2018) [58] | Demonstration of imaging interpretation strategy |  |  |  |  |  |  | * |  |  |  |  |  |
|  | Stanton (2020) [59] | 20 hours of in-depth training from Pain Science Education Expert |  |  |  |  |  |  | * |  |  |  |  |  |
| Prompts/  Cues | Karran (2018) [58] | Text message follow-up prompting to display/read the relevant poster |  |  | * |  |  | *** |  |  |  |  |  |  |
| Behavioural Practice/Rehearsal | Karran (2018) [58] | Practice and rehearsal of imaging interpretation strategy |  |  |  |  |  |  | ** |  |  |  |  |  |
|  | Stanton (2020) [59] | training from Pain Science Education Expert including practice and rehearsal |  |  |  |  |  |  | ** |  |  |  |  |  |
| Credible Source | Karran (2018) [58] | Provision of video resource with expert in the field presenting |  |  |  |  |  |  |  |  |  |  |  |  |
| Avoidance/reducing exposure to cues for the behaviour | Ash (2008) [17] | Patient and clinicians blinded to MRI results |  |  |  |  |  |  |  |  |  |  |  |  |
|  | Karran  (2017) [48] | Online simulation:  best practice care arm does not receive a lumbar MRI scan |  |  |  |  |  |  |  |  |  |  |  |  |
|  | Bossen (2013) [61] | MRI reports reworded using neutral wording and optimistic interpretations |  |  |  |  |  |  |  |  |  |  |  |  |
|  | Rajasekaran (2021) [60] | Alternate method of reporting designed to avoid concerning terminology |  |  |  |  |  |  |  |  |  |  |  |  |
| Adding objects to the environment | Karran (2018) [58] | Use Visual aids, take home resources, & links to online information |  |  |  |  |  | * |  |  |  |  |  |  |
|  | Stanton (2020) [59] | Use of intervention resources (explain pain book, news story) |  |  |  |  |  | * |  |  |  |  |  |  |
|  | Karran  (2017) [48] | Addition of pre-information about scan findings |  |  |  |  |  |  |  |  |  |  |  |  |
|  | Zhuang (2019) [62] | Use of 3D printed model based on imaging findings to explain condition |  |  |  |  |  | * |  |  |  |  |  |  |
| Framing/Reframing | Bossen (2013) [61] | MRI reports reworded using neutral wording and optimistic interpretations |  |  |  |  |  |  |  |  |  |  |  |  |
|  | Fried (2018) [54] | Addition of prevalence statement |  |  |  |  |  |  |  |  |  |  |  |  |
|  | Jarvik (2020) [55] | Addition of prevalence statement |  |  |  |  |  |  |  |  |  |  |  |  |
|  | Karran (2018) [58] | Re-interpretation of imaging findings highlighting ‘positive’ features |  |  |  |  |  |  |  |  |  |  |  |  |
|  | Medalian (2019) [56] | Addition of age specific prevalence information |  |  |  |  |  |  |  |  |  |  |  |  |
|  | Karran  (2017) [48] | Addition of age specific prevalence information |  |  |  |  |  |  |  |  |  |  |  |  |
|  | Rajasekaran (2021) Phase I [60] | Reassurance only incidental and age-related findings |  |  |  |  |  |  |  |  |  |  |  |  |
|  | Rajasekaran (2021)  Phase III [60] | reporting designed to avoid concerning/catastrophising terminologies |  |  |  |  |  |  |  |  |  |  |  |  |
|  | Stanton (2020) [59] | Use of intervention resources (explain pain book, news story) |  |  |  |  |  |  |  |  |  |  |  |  |
|  | Weeks (2020) [57] | Addition of epidemiological information in lumbar MRI reports |  |  |  |  |  |  |  |  |  |  |  |  |
| Incompatible beliefs | Fried (2018) [54] | statement regarding prevalence of common findings |  |  |  |  |  |  |  |  |  |  |  |  |
|  | Jarvik (2020) [55] | Addition of benchmarks indicating prevalence of common findings |  |  |  |  |  |  |  |  |  |  |  |  |
|  | Karran (2018) [58] | Discussion about prevalence of degenerative features in asymptomatic adults |  |  |  |  |  |  |  |  |  |  |  |  |
|  | Medalian (2019) [56] | Addition of age specific prevalence information to MRI reports |  |  |  |  |  |  |  |  |  |  |  |  |
|  | Karran  (2017) [48] | Addition of age specific prevalence information |  |  |  |  |  |  |  |  |  |  |  |  |
|  | Stanton (2020) [59] | Highlighting stages of OA and % without pain |  |  |  |  |  |  |  |  |  |  |  |  |
|  | Weeks (2020) [57] | Addition of epidemiological information in lumbar MRI reports |  |  |  |  |  |  |  |  |  |  |  |  |
| * - p<0.01 ** - p = 0.013 *** - p=0.04  P values indicating a strength of association between the BCT and their mechanism of action [49] | | | | | | | | | | | | | | |
