# Supplementary material for: Evaluating musculoskeletal imaging communication interventions using behavioural science: a scoping review using the COM-B model
Source: BMJ Open. 2025 Apr 9;15(4):e085807. doi: 10.1136/bmjopen-2024-085807 (PMC11987107; doi:10.1136/bmjopen-2024-085807)
Supplement: Supplementary file 2 [file bmjopen-2024-085807.reviewer_comments.pdf]

PEER REVIEW HISTORY

BMJ Open publishes all reviews undertaken for accepted manuscripts. Reviewers are asked to complete a checklist review form and are provided with free text boxes to elaborate on their assessment. These free text comments are reproduced below.

ARTICLE DETAILS

Title (Provisional)

Evaluating Musculoskeletal imaging communication interventions using behavioural science: A Scoping Review using the COM-B Model

Authors

Kirby, Edward; MacMillan, Andrew; Brinkley, Andrew; X.W. Liew, Bernard; Bateman, Andrew

---

VERSION 1 - REVIEW

**Reviewer**	**1**
**Name**	**Dean, Sarah**
**Affiliation**	**PenCLAHRC University of Exeter Medical School**
**Date**	**01-Nov-2024**
**COI**	**None**

BMJ open Kirby et al Nov2024

This is a great piece of work and exciting to see. However, it is also a challenging paper to read because of the level of technical understanding required to follow elements of the COM-B model, Theoretical Domains Framework, Behaviour Change Wheel and Behaviour Change Techniques (BCT) taxonomy. As a behavioural scientist and someone who is very familiar with the BCT taxonomy I believe that this is an important and useful paper, but it will need to be made more accessible to non-specialists, it took me at least 3 reads to follow! I am not entirely sure how to recommend making it an easier read, but I would want the authors to attempt simplifying. This this might mean putting some of the more detailed information into separate supplementary files or perhaps having explanatory boxes to separate out some of the jargon heavy text.

My only other major concern is that the ‘strengths and limitations’ statements pick up on what I believe is the greatest limitation of this work without really having that fully expressed in the discussion. I believe a statement needs to be inserted into the discussion about the fact that there may be more studies that do involve theoretically informed communication about imaging for musculoskeletal patients, but they were embedded in a more comprehensive treatment or management intervention. Thus, they were missed by this search strategy because you were only looking for communication interventions specifically. Therefore, to say that no evidence exists for theoretically informed communication is possibly an overstatement - it doesn't exist in

the papers you reviewed because the only ones you found that did include theory were the two feasibility studies and none were effectiveness studies.

There are several minor amendments and suggestions for editing listed below.

Page 2 in the abstract line 23. Stated that search was from inception to the current day, but this ‘current day’ has now passed, so it may be better to put the actual date that your search was completed.

Page 3 lines 4 to 6. This is a very long sentence and could be improved by splitting. The abbreviation MSK should perhaps also the written in full at the start of the main manuscript.

Line 23 is a little confusing because you started by saying increased costs and then go on to talk about low value treatments. I think here you mean low therapeutic value treatments rather than low value in terms of their cost?

Line 24 you state that clinical guidelines recommend addressing such miss held beliefs in all patients. This suggests all patients hold miss held beliefs, but this may not be true? Perhaps it is better to say guidelines recommend checking for miss held beliefs in all patients? Of course the guidelines may have written it as you have it, which case it is perhaps better just to qualify the recommendation.

Line 33 to 34 you put willingness to maintain the patient clinician relationship. I can guess what you mean by this, but it is not an explicitly clear statement and perhaps needs reworking.

Page 3 line 57 – insert ‘it helps to create’ to ‘inform intervention design in multiple healthcare settings, demonstrating it helps to create acceptable, feasible, effective….’ I suggest adjusting the wording of this sentence to help clarity.

Page 4 line 4: does this need a reference.

Line 29 Fourth bullet point - to interpret the proposed mechanism of action of each BCT. This seems a bit ambitious as I did not see all 41 identified BCTs listed anywhere (only 11 in Table 2). Did you mean proposed mechanism of action for a BCT group (cluster)? See also later point.

Page 7 line 4. It would be helpful to insert ‘feasibility’ between ‘two studies’. It would be also really helpful to have reference numbers inserted into the table so that when we see the lead author name we also see the same reference number. It took a little while to work out that Karran et al 2018 was reference 53. Having the name and number together each time would really help the reader navigate around your paper and tables.

Line 98. Should this be ‘introduced’ not ‘introduce’?

Line 11. Rather than it being another feasibility study surely it is the second feasibility study?

Line 16. Insert ‘also’? ‘and for this reason this study was also categorised as testing theory’.

Line 17 you say that all other studies in this review do not cite a specific theory or rationale but I'm not sure I agree. Some of them did have a rationale, even though these were not necessarily underpinned by a specific theory. The Tidier checklist does actually say rationale or theory or goal, and it looks as if you have indicated authors’ proposed rationales even if they are not specific theories.

Line 20 adjust this, so it includes ‘rationales’? Otherwise, lines 17-20 are a bit confusing.

Line 25 here you talk about the BCT's (so individual units of change) but also the categories of BCT's (which in the taxonomy are actually called clusters or groups and not categories). I don't really mind what term is used but I do wonder if you need to include this detail. Does it add to the understanding of the paper? Perhaps it does because you only report 11 BCTs in Table 2 (see earlier point). Perhaps make a decision about what level to report to: individual BCTs and n=41 (of 93) were used, or BCT cluster, n=11 (of 16) were used, and you have just listed the most commonly used BCTs (from the cluster) in Table 2?

Page 8 Table 1 It would be helpful to have the reference numbers added so that it's much easier to cross refer to the discussion to the text.

Lines 36 to 40 not clear. In line 36 you say three of the six COM-B components are used and three were not targeted. Later say only 2 studies targeted all three COM-B components. The solution here is to say that a total of three out of the six COM-B components were targeted by included studies, only two studies used all of these three components, and the three remaining components were not targeted at all. Does this make more sense?

Table 2 at the end of the table you have some *P* values and then a footnote explaining the *P* values. None of this was mentioned in the methods, what was the hypothesis, how was it analysed, is it a powered analysis etc. What are you trying to show here? Given the length of the paper and the complexity of the paper could this be reported elsewhere? If not then more information is needed, perhaps in supplementary files.

Page 10 line 42. The abbreviation PSE may have already been explained but if not it needs to be in full here.

Line 50. States ‘and then complete their exercise plans’ - this could be confused with carrying out their exercise, should ‘complete’ be replaced with ‘prepare’? That way it would be fully congruent with your coding as the Action Planning BCT.

Page 11 discussion section. Line 38 you state currently no evidence to support the effectiveness of utilising theory when developing imaging reporting interventions and I do wonder if the issue is that in the studies you have reviewed there is no evidence of effectiveness rather than saying overall there is no evidence. This is because there are other studies that have embedded the type of communication intervention that is of interest to you but were not picked up by your search strategy (as you hint in the bullet points). I don't think this is a fatal flaw, but I do think it needs to be made clearer that this study only searched for/found communication interventions that had a sole focus on reporting imaging; and not all communication interventions that contain attempts to address the misunderstanding of imaging were found because these messages were only part of their much broader intervention.

End of page 12. Are you being a little bit harsh? Some of the studies included in your review would have been designed before the BCT Taxonomy was published, and certainly before it became well known. The BCT taxonomy is key here as it enables the operationalisation of theory; so whilst this doesn’t excuse studies that completely failed to consider theory in their intervention design it is perhaps understandable....

Page 13 line 57. Please include which study reference number at the start of this sentence (is it 4) as this would be helpful.

Page 14 line 26. Spelling error of ‘complements’.

Line 60. This is a slightly obscured way of saying there may be some better studies out there that were missed by the search strategy. This statement needs to be more explicit and/or something put into the discussion (see earlier point).

Page 15 line 1. The final bullet point suggests the accuracy of coding is only determined by the reporting. In my view the accuracy of coding is affected by both the reporting but also how well coders interpret the taxonomy, which is sometimes not easy to agree upon.

Page 15 line 24. Should be data ‘were’, not data ‘was’.

Reference list. There are numerous errors in the list that will need addressing. There are missing details, use of first name of authors and other formatting inconsistencies. From page 27 onwards a very large number of references have no journal source.

In summary an interesting paper and analysis, important to report these findings. I trust my suggestions help refine the paper so that it becomes accessible and useful to a wider audience.

**Reviewer**	**2**
**Name**	**Creaser, Amy**
**Affiliation**	**University of Leeds**
**Date**	**09-Dec-2024**
**COI**	**None**

WHAT BEHAVIOUR CHANGE TECHNIQUES HAVE BEEN USED TO AFFECT THE COMMUNICATION OF MUSCULOSKELETAL IMAGING? A SCOPING REVIEW USING THE COM-B MODEL.

This scoping review investigates the mechanisms of action of interventions targeting the communication of musculoskeletal imaging results. At present, I would not recommend this paper for publication. This review has potential to be insightful and could be used to aid the development of future interventions. However, the findings are descriptive and does not link the mechanisms of action (BCTs, COM-B components) with intervention effectiveness. Including this would be more useful for readers and allow this review to inform intervention development. There were also some elements that were vague, such as the aims and methodology.

Title

I find the title vague. There is no reference to interventions/feasibility studies etc. Behaviour change techniques were not the only thing coded in this review. Should it be stated that interventions are around how a professional communicates the images?

Abstract

“A detailed analysis of the interventions chosen by researchers has not been performed to date” –I am not sure what is meant by “chosen by researchers”. This could be removed.

No aims or research questions are included in the abstract.

Methods section of the abstract doesn't give any indication of the inclusion/exclusion criteria.

Results – an overview of the study designs could be provided, rather than just listing the BCTs. What about the effectiveness of the interventions?

Strength and limitation of this study

"**Validated** behaviour change framework" is an overstatement. I would remove 'validated'.

When first reading "the quality of the study intervention reporting determined the accuracy of the coding" it appeared that a quality assessment/risk of bias was conducted for the studies included in the review. However, this was not conducted. I would reword this statement to remove the word quality.

Introduction

Line 9, "A reason cited for these rising costs and disability levels is the perpetuation of negative or unhelpful beliefs about the nature of MSK conditions" – An interesting point. It would be good to expand, who claims this?

Line 13, "The belief, based on the biomedical model of health, that abnormalities visible on imaging are causally linked with pain [34] has been associated with negative consequences to patients and healthcare systems [34]" – It would be good to expand, what negative consequences?

Lines 36 and 37 – The final sentence could be expanded. I think it needs to be made clear that understanding barriers is important for designing interventions, which would nicely link with the next paragraph. Also, it would be useful to include the importance of intervening here – for patient, economical outcomes for example.

Line 49 onwards. You discuss COM-B here, but it would also be good to include BCTs. Include how the COM-B model and BCTTv1 are interconnected and the value of coding for both.

Aims

The aims are not specific and are quite vague and ambiguous.

You haven't **defined** which BCTs, you have **identified** the BCTs.

Mentioned briefly how you will address the aims, as they are currently vague – e.g. "chart, "interpret".

The final aim mentions mechanisms of action, but this terminology isn't mentioned in the introduction before this aim. This needs mentioning earlier and also defining.

Methods

Eligibility criteria – why was Participants, Concept and Context (PCC) criteria not used given it is a scoping review and followed JBI methodology?

As above, it is unclear to me who the participants are in this review. Did studies have to include professionals to communicate results?

How were non-English studies translated? Using a software? A member of the research team?

Were only peer-reviewed academic studies included? Grey literature? In the information sources and search strategy section you mention that the strategy aims to locate published studies. This needs to be made clear in the eligibility criteria section.

Information sources and search strategy – This section needs a bit of work and rearranging. Was the search conducted on the 9th February the only search conducted or was this an updated search? The three-steps need to also be made clearer – what were these and what was done at each step?

Study selection – Expand on what was conducted in the 'pilot test' – how practically was this done? Provide enough information so this could be replicated. Similarly, 'assessment against the inclusion criteria' – how was this practically conducted?

Could a copy of the data extraction form be included as a supplementary material?

Data analysis – The fact authors were conducted due to lack of information/data needs to be made clear in the study selection section.

How was the coding manual developed?

Need to make it clear whether risk of bias/quality assessment was undertaken. This isn't required for a scoping review so it is perfectly fine if it wasn't, but a sentence stating this would be useful.

Results

Line 52, "One further eligible study was identified by manual searching" – it needs to be made clearer in the methods section how this was conducted – was it via a reference list for example?

PRISMA diagram – the final results are confusing, it appears like a total of 22 studies were included. Is the 'reports of included studies' necessary? Similarly, "reasons 1-6" – can the reasons not be included directly in the diagram?

More information on what the interventions look like should be included. This also isn't included in supplementary table 3. It is more useful for readers if they have an understanding of the interventions before understanding the mechanisms of action. For example, if you were reading this review to support the design of a new intervention you would want to have an understanding of what the interventions did.

Can supplementary table 3 be included in the main text of the paper?

In the 'Use of theory and/or systematic intervention design process' there is mention of BCTs in a feasibility study. Is this appropriate in this section? Is this section only including information for those that have explicitly reported using theory or BCTs? (e.g. not the ones you as authors of the review have coded?). I found this section a bit unclear.

There is no reference to the effectiveness of the studies, even though the results are provided in supplementary table 3. The effectiveness of the interventions should be included in text to provide an understand of the effectiveness of the BCTs, targeted COM-B components. Again, imagine you are reading this review to support your own intervention development and consider what you would find useful.

Discussion

You mention how BCTs not used in the included studies have successfully changed behaviour, but, in the results, you do not discuss the effectiveness of the BCTs they do use.

Expand on the strengths and limitations of the review. I would recommend acknowledging the behaviour change technique ontology. This has since been published since you conducted the BCT coding. This include more BCTs than the BCTTv1, therefore may not be as comprehensive as it would have been if this ontology was already published - [The Behaviour Change Technique Ontology: Transforming the Behaviour Change Technique Taxonomy v1 - PMC]

VERSION 1 - AUTHOR RESPONSE

Reviewer 1 comments

Suggested Amendment	Response	Location in text
**Paper needs to be made more accessible to non-specialists**	Complex data moved to supplementary files and simplified summaries provided in main manuscript. Jargon replaced with understandable terms throughout. Description of how BCT relates to COM-B target simplified.	
**Page 2 in the abstract, line 23: Stated that search was from inception to the current day, but this 'current day' had passed. Suggested stating the actual date of completion.**	The actual date of search completion was inserted to provide clarity.	Pg 2 line 30
**Page 3, lines 4 to 6: Sentence was too long and needed splitting. The abbreviation MSK should have been written in full at first mention.**	The sentence was revised for clarity and split into two. 'Musculoskeletal (MSK)' was spelled out in full at its first mention.	Pg 4 line 7
**Line 23: The phrase 'low value treatments' was unclear. Suggested 'low therapeutic value treatments' to avoid confusion.**	'Low value treatments' was revised to 'treatments of low therapeutic value treatments' to improve accuracy.	Pg 4 line 29
**Line 24: Stated that clinical guidelines recommend addressing 'miss held beliefs' in all patients, which implied all patients had them.**	The sentence was reworded to 'identifying and addressing such unhelpful beliefs in patients presenting with MSK symptoms' to reflect that presence of such beliefs in this group of patients.	Pg 4 line 29
**Lines 33 to 34: The phrase 'willingness to maintain the patient-clinician relationship' was unclear.**	The phrase was revised to 'reluctance to challenge the patients' current beliefs about the cause of pain'.	Pg 4 line 40
**Page 3, line 57: suggest adjusting the wording of this sentence to help clarity**	The sentence was significantly adjusted.	Pg 5 line 33
**Page 4, line 4: Suggested adding a reference.**	A relevant reference was added to support the the rising burden of MSK disability and health system costs.	Pg 6 line 7

**Line 29, fourth bullet point: Interpreting the proposed mechanism of action for all 41 BCTs seemed ambitious since only 11 were listed in Table 2. Suggested clarifying whether mechanisms applied to individual BCTs or clusters.**	This objective was altered to 'Determine which behavioural components (Capability, Opportunity, Motivation) these BCTs target to change clinician communication behaviour.'	Pg 6 line 35
**Page 7, line 4: Suggested inserting 'feasibility' between 'two studies'. Also recommended adding reference numbers to the table for easier navigation.**	Sentence rewritten	Pg 10 line 25
**Line 98: Suggested changing 'introduce' to 'introduced'.**	'Introduce' was corrected to 'introduced'.	Pg 10 line 31
**Line 11: Suggested 'second feasibility study' rather than 'another feasibility study'.**	The phrase was revised to 'second feasibility study'.	Pg 10 line 36
**Line 16: Suggested inserting 'also' before 'categorised as testing theory'.**	'Also' was added to improve sentence flow.	Pg 10 line 41
**Line 17: Suggested clarifying that some studies had rationales even if they were not underpinned by a specific theory.**	Sentence reworded and relocated	Pg 11 line 2
**Line 20: Suggested including 'rationales' to align with previous clarifications.**	n/a as sentence removed	
**Line 25: Suggested reviewing the terminology for BCT categories/clusters and deciding on the most appropriate level of reporting.**	Level of reporting amended to individual BCT's and their COM-B components	
**Page 8, Table 1: Suggested adding reference numbers for easier cross-referencing.**	Reference numbers were added to Table 1.	Pg 11
**Lines 36 to 40: Suggested rewording to clearly state how many COM-B components were targeted and how many studies used all three targeted components.**	The section was revised to 'A total of 3 out of the 6 COM-B components were targeted' and 'Two feasibility studies targeted three COM-B components simultaneously' to add clarity.	Pg 13 line 4
**Table 2: P-values and their explanation were not mentioned in the methods. Suggested either providing more detail or reporting elsewhere.**	Additional information was included in the methods to explain the provenance of the p values.	Pg 7 line 6
**Page 10, line 42: Suggested defining PSE abbreviation if not previously explained.**	'PSE' was defined in full at first use.	Pg 10 line 36

**Line 50: Suggested replacing ‘complete’ with ‘prepare’ to better align with Action Planning BCT coding.**	‘Complete’ was replaced with ‘prepare’ to improve clarity.	
**Page 11, discussion section, line 38: Suggested clarifying that the review found no evidence of effectiveness rather than stating no evidence existed at all.**	The text was extensively revised as part of another revision to add effectiveness data.	Pg 15 line 16
**End of page 12: Suggested softening critique of studies designed before the BCT Taxonomy was well known.**	A paragraph was added to acknowledge that there have been considerable advancements in the field of complex intervention research since many of the studies published	Pg 18 line 4
**Page 13, line 57: Suggested including the reference number at the start of the sentence for clarity.**	The reference number was added.	Pg 22 line 13
**Page 14, line 26: Spelling error ‘complements’.**	The spelling error was corrected.	Pg 22 line 31
**Line 60: Suggested making it clearer that the search strategy may have missed some relevant studies.**	A paragraph was added explaining this limitation	Pg 23 line 12
**Page 15, line 1: Suggested clarifying that coding accuracy is influenced by both reporting quality and coder interpretation.**	The text was revised to reflect subjectivity of coding	pg 23 line 24
**Page 15, line 24: Suggested changing ‘data was’ to ‘data were’.**	The grammatical correction was made.	Pg 24 line 38
**Reference list: Numerous errors, missing details, and inconsistencies were noted.**	The reference list was thoroughly reviewed and corrected for consistency and completeness.	

Reviewer 2 comments

Suggested amendment	Response	Location in text
**Title: The title was vague, lacking reference to interventions/feasibility studies. Suggested specifying that interventions relate to professional communication of imaging.**	The title was revised to clarify its focus on interventions related to professional communication of imaging.	
**Abstract: The phrase 'chosen by researchers' was unclear. Suggested removal.**	The phrase was removed.	

**No aims or research questions included in the abstract.**	Aims and research questions were added to the abstract and reformatted according to the editor comments	Pg 2 line 20
**Methods section of the abstract lacked inclusion/exclusion criteria.**	Summary of Inclusion and exclusion criteria were added to the abstract.	Pg 2 line 32
**Results section listed BCTs but did not provide an overview of study designs or intervention effectiveness.**	A summary of effectiveness findings was added and Study designs detailed	Pg 3 line 7
**Strength and limitations: 'Validated behaviour change framework' was an overstatement. Suggested removing 'validated'.**	Sentence removed as per editor comments.	Pg 3 line 24
**The phrase 'quality of the study intervention reporting determined the accuracy of the coding' suggested a quality assessment was performed when it was not. Suggested rewording.**	This limitation was removed and replaced with statement about the subjectivity of coding	Pg 3 line 32
**Introduction: Line 9, 'A reason cited for these rising costs...' should specify who claims this.**	A citation was added to clarify the source of the claim.	Pg 4 line 12
**Line 13, negative consequences of imaging beliefs should be expanded.**	Additional details on the negative consequences were included.	Pg 4 line 36
**Lines 36-37: The final sentence should highlight the importance of understanding barriers for intervention design and patient/economic outcomes.**	The start of the following sentence was changed to link the barriers with reasons for understanding barriers	Pg 5 line 16
**Line 49 onwards: Discussion of COM-B should include BCTs and their interconnection.**	The section was revised to include a discussion of BCTs and their link to COM-B including an example.	Pg 5 line 43
**Aims: The aims were vague.**	The aims were rewritten to make them more clear and specific	Pg 6 line 25
**The term 'mechanisms of action' appeared in the aims but was not introduced in the introduction.**	The term mechanism of action was removed. Level of reporting altered according to previous reviewer comments	Pg 6 line 35
**Methods: Eligibility criteria should justify why PCC was not used.**	PCC was added to describe how search terms were developed: target population (adults with MSK conditions), concept (communication of imaging) and context (experimental studies in any setting worldwide.	Pg 7 line 6

**Clarification needed on whether studies had to include professionals communicating results.**	The eligibility criteria were revised to include patients and clinicians for clarity.	Pg 7 line 31
**Non-English study translation process was unclear.**	This statement was removed as no eligible non-english studies were identified	
**The inclusion of grey literature was unclear.**	The eligibility criteria explicitly stated whether grey literature was included.	Pg 7 line 12
**Information sources and search strategy required clarification and rearrangement.**	The section was restructured for clarity, and the search process was explained step-by-step.	Pg 7 line 4
**Study selection: More detail was needed on the pilot test and inclusion assessment.**	The pilot test process and inclusion assessment were expanded.	Pg 8 line 15
**Suggested including a copy of the data extraction form in supplementary materials.**	a copy of the data extraction form was included in the supplementary materials (table 2)and referenced in the manuscript	Pg 8 line 24
**Data analysis: Clarify that authors were contacted due to data gaps.**	This clarification was added to the study selection section.	Pg 8 line 38
**The coding manual development process was unclear.**	The process for developing the coding manual was explained.	Pg 9 line 7
**Clarify whether a risk of bias/quality assessment was conducted.**	A statement was added to clarify that no quality assessment was performed.	Pg 9 line 30
**Results: Line 52, manual searching process needed more detail.**	The process for manual searching was clarified in the information and search strategy section.	Pg 7 line 12
**PRISMA diagram appeared to indicate 22 studies; 'reports of included studies' might be unnecessary. Suggested including exclusion reasons directly in the diagram.**	The PRISMA diagram was revised for clarity, and exclusion reasons were incorporated.	Supplementary figure 1
**More details on interventions were needed before discussing mechanisms of action.**	A description of interventions was added to Table 1and 2 and the TiDIER.	Supplementary table 5
**'Use of theory and/or systematic intervention design process' section needed clarification regarding feasibility studies and coded BCTs.**	The section was revised to define which study was being discussed and whether it was a feasibility trial	Pg 10 line 19
**The results did not discuss intervention effectiveness.**	A summary of intervention effectiveness was added to the results section. Standardised Mead Differences calculated and forest plots developed (Supplementary figure 2)	Pg 16 line 15

**Discussion: Effectiveness of BCTs used in the included studies was not discussed.**	The discussion was expanded to address the effectiveness of BCTs used.	Effectiveness discussed in each COM-B heading within discussion eg. pg 20 line 6
**Strengths and limitations: Suggested acknowledging the Behaviour Change Technique Ontology, which was published after the study.**	The ontology was acknowledged, with a note on its expanded BCT coverage.	Pg 23 line 5
